# CAR-T cell immunotherapy for ovarian cancer: hushing the silent killer

**DOI:** 10.3389/fimmu.2023.1302307

**Published:** 2023-12-07

**Authors:** Fatemeh Nasiri, Khadijeh Farrokhi, Pouya Safarzadeh Kozani, Maral Mahboubi Kancha, Setareh Dashti Shokoohi, Pooria Safarzadeh Kozani

**Affiliations:** ^1^ Department of Medical Biotechnology, Faculty of Medical Sciences, Tarbiat Modares University, Tehran, Iran; ^2^ Department of Production Platforms & Analytics, Human Health Therapeutics Research Centre, National Research Council Canada, Montreal, QC, Canada; ^3^ Department of Microbial Biotechnology, Faculty of Biotechnology, Amol University of Special Modern Technologies, Amol, Iran; ^4^ Department of Medical Nanotechnology, School of Medicine, Shahroud University of Medical Sciences, Shahroud, Iran

**Keywords:** cancer immunotherapy, chimeric antigen receptor, ovarian cancer, solid tumors, adoptive cell therapy

## Abstract

As the most lethal gynecologic oncological indication, carcinoma of the ovary has been ranked as the 5^th^ cause of cancer-related mortality in women, with a high percentage of the patients being diagnosed at late stages of the disease and a five-year survival of ~ 30%. Ovarian cancer patients conventionally undergo surgery for tumor removal followed by platinum- or taxane-based chemotherapy; however, a high percentage of patients experience tumor relapse. Cancer immunotherapy has been regarded as a silver lining in the treatment of patients with various immunological or oncological indications; however, mirvetuximab soravtansine (a folate receptor α-specific mAb) and bevacizumab (a VEGF-A-specific mAb) are the only immunotherapeutics approved for the treatment of ovarian cancer patients. Chimeric antigen receptor T-cell (CAR-T) therapy has achieved tremendous clinical success in the treatment of patients with certain B-cell lymphomas and leukemias, as well as multiple myeloma. In the context of solid tumors, CAR-T therapies face serious obstacles that limit their therapeutic benefit. Such hindrances include the immunosuppressive nature of solid tumors, impaired tumor infiltration, lack of qualified tumor-associated antigens, and compromised stimulation and persistence of CAR-Ts following administration. Over the past years, researchers have made arduous attempts to apply CAR-T therapy to ovarian cancer. In this review, we outline the principles of CAR-T therapy and then highlight its limitations in the context of solid tumors. Ultimately, we focus on preclinical and clinical findings achieved in CAR-T-mediated targeting of different ovarian cancer-associated target antigens.

## 1 Introduction

**Figure 1:**
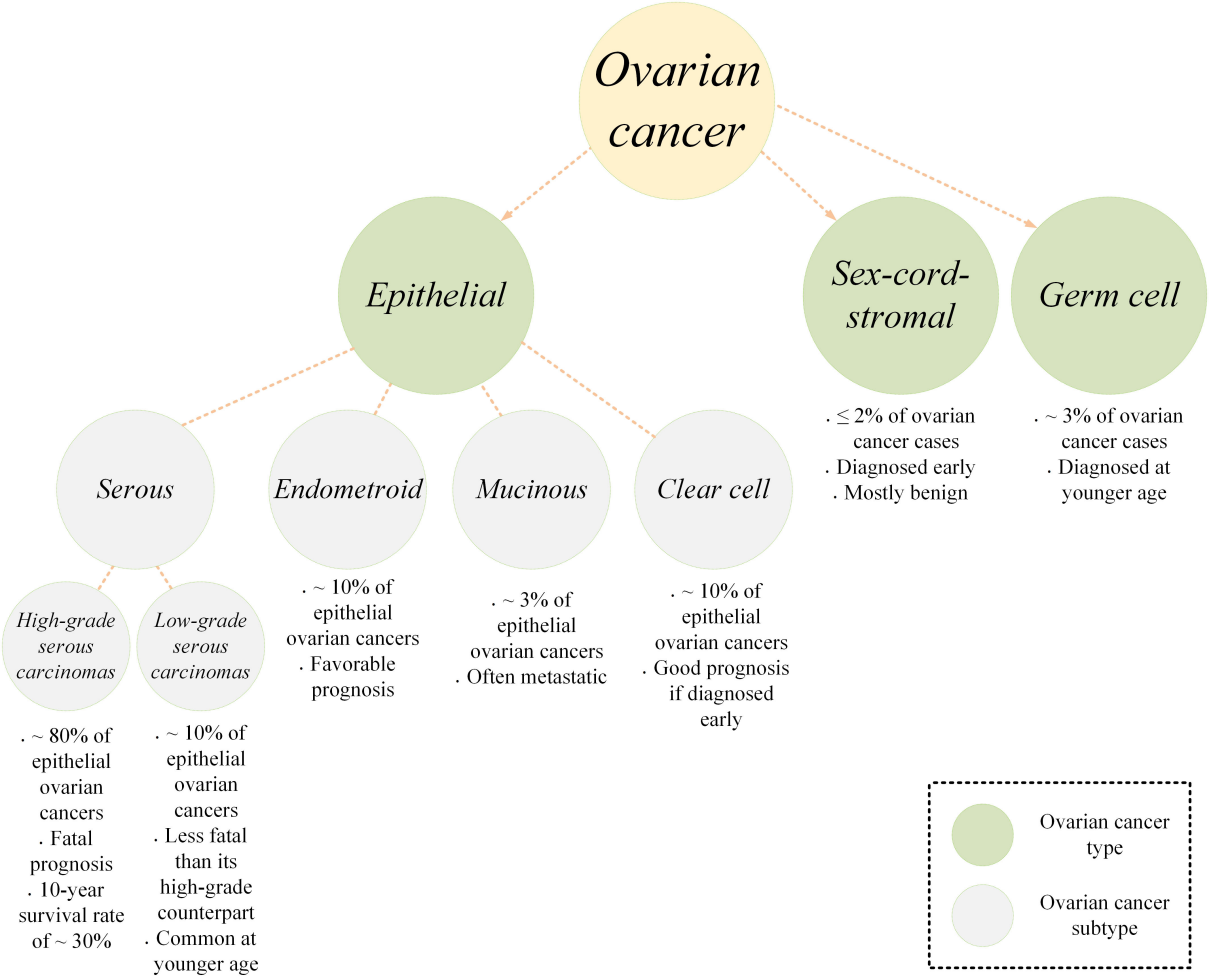
A simplified schematic of the ovarian cancer types and subtypes.

Ovarian cancer is a type of malignant tumor involving the ovary tissue. It is originally derived from the ovary itself but it can also originate from other structures in the vicinity of an ovary including the fallopian tubes (1, 2). Ovarian cancer is the most fatal gynecologic neoplasm (3). This often called “*silent killer*” type of cancer is among cancers with relatively poor prognosis mainly because it is not accurately diagnosed until it reaches its late and advanced stages generally due to its vague and/or common clinical symptoms (3, 4). Statistics indicate that more than 70% of ovarian cancer cases are not diagnosed before stage III or IV, and the five-year survival rate for patients is reported to be around 47% (5). According to estimations, around 19,000 new cases of ovarian cancer are diagnosed annually and around 12,000 ovarian cancer-related mortality occur each year (6). In terms of classification, ovarian cancer is categorized into three main types, epithelial, germ cell, and sex-cord-stromal (**Figure 1**) (7). Epithelial ovarian cancer is the most common type among the diagnosed cases accounting for around 95% of all the cases. This type has four subtypes including serous, endometrioid, mucinous, and clear cell. Serous epithelial ovarian cancer is also categorized into high-grade serous carcinomas (HGSC) or low-grade serous carcinomas (LGSC). HGSC is the most common subtype of epithelial ovarian cancer accounting for around 70%. This is while LGSC, endometrioid, mucinous, and clear cell account for around 5, 10, 3, and 10%, respectively (7). The standard of care for the treatment of ovarian cancer is surgery, radiation therapy, and chemotherapy using platinum-based chemotherapeutic agents (8–10). Such treatment modalities can only be clinically beneficial in patients diagnosed with early-stage diseases. In the case of late-stage diagnosis, patients will experience refectory or recurrent disease, and disease-free intervals will be shorter in such cases.

Accumulating evidence indicates that tumor-infiltrating lymphocytes (TILs) have remarkable clinical significance and prognostic value in various types of solid tumors, revealing their immunogenic nature (11–14). Ovarian cancer is also among malignancies in which TILs play an important role in the clinical response and clinical outcomes of the patients. In detail, there are statistically significant differences in the distributions of overall survival (OS) and progression-free survival (PFS) in ovarian cancer patients based on the presence or absence of TILs (15). According to a study, the five-year OS rate for ovarian cancer patients with and without TILs was 38 and 4.5%, respectively (15). Moreover, it has been reported that the five-year OS rate for ovarian cancer patients with a complete response after chemotherapy using platinum-based agents or debulking was 73.9 and 11.9% in patients with and without TILs, respectively (15). Research findings also indicate that ovarian cancer patients with TILs exhibit increased intratumoral expression of INF-γ, IL-2, and T-cell-associated chemokines alongside having postponed disease recurrence and death while patients with no TIL have profiles of elevated levels of vascular endothelial growth factor (VEGF) expression (15). Such findings point out the fact that ovarian cancer has an immunogenic nature; therefore, immune-based therapies could be leveraged as potent treatment modalities.

**Figure 2:**
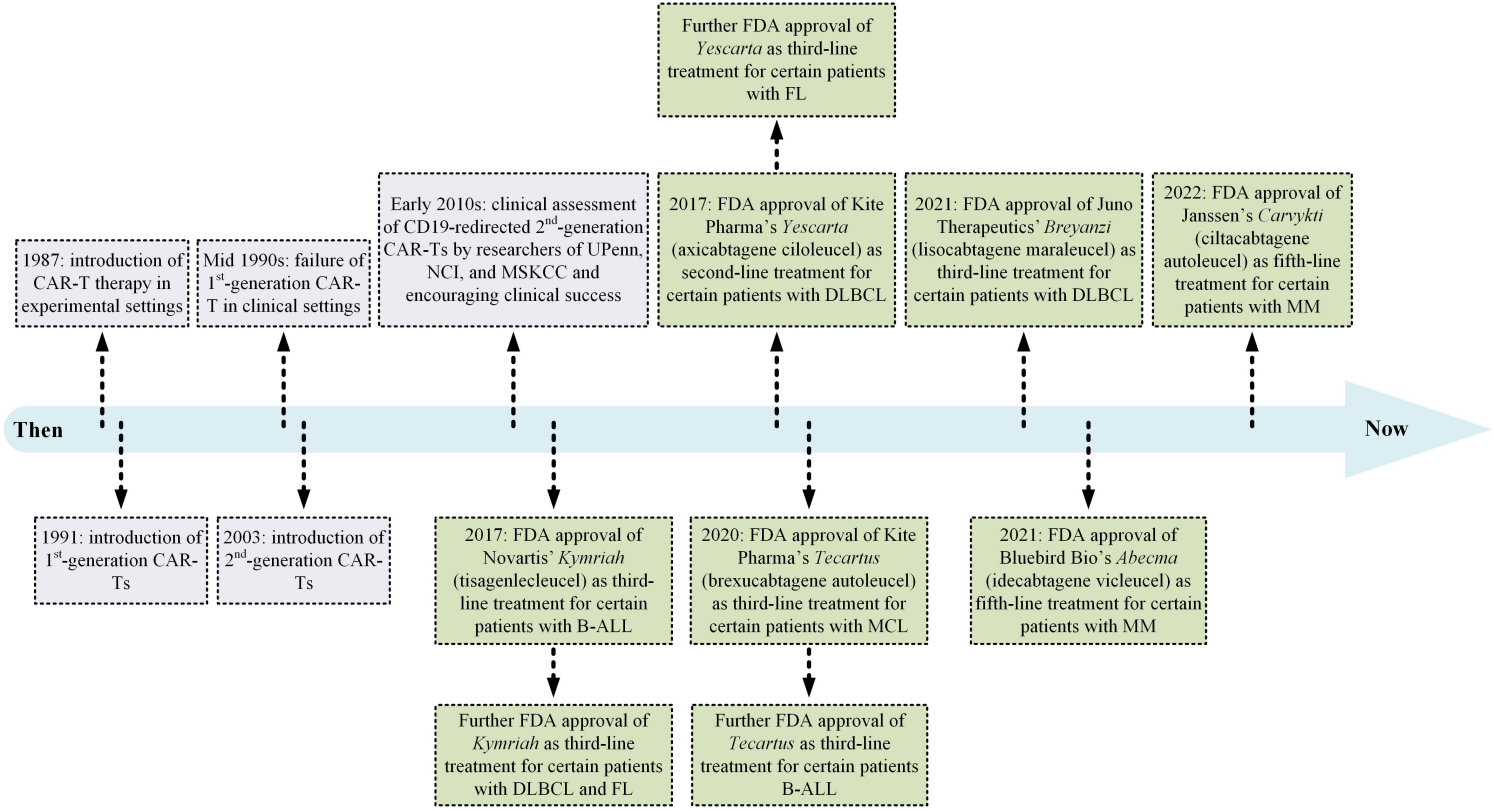
A detailed timeline of CAR-T therapy development from the laboratory bench to the approval of several products by the US FDA for medical use. B-ALL, B-cell acute lymphoblastic leukemia; DLBCL, diffuse large B-cell lymphoma; FDA, Food and Drug Administration; FL, follicular lymphoma; MCL, mantel cell lymphoma; MM, multiple myeloma; MSKCC, Memorial Sloan Kettering Cancer Center; NCI, National Cancer Institute; UPenn, University of Pennsylvania.

Cancer immunotherapy has given hope to patients with advanced oncological and immunological indications over the past 40 years. Since then, multiple platforms of this highly effective treatment strategy have been established, and the United States Food and Drug Administration (US FDA) has granted permission to a large number of immunotherapeutics for medical use. Monoclonal antibodies (mAbs; with the first approval in 1986), T-cell-redirecting bispecific antibodies (TRBAs; with the first approval in 2014), antibody-drug conjugates (ADCs; with the first approval in 2000), and chimeric antigen receptor T lymphocytes (CAR-Ts; with the first approval in 2017) are examples of how cancer immunotherapy has changed the landscape of cancer treatment in an effective and targeted fashion (16–19). To date, six CAR-T products (**Figure 2**) and more than a hundred mAbs have been given the green light for clinical use by the US FDA.

**Figure 3:**
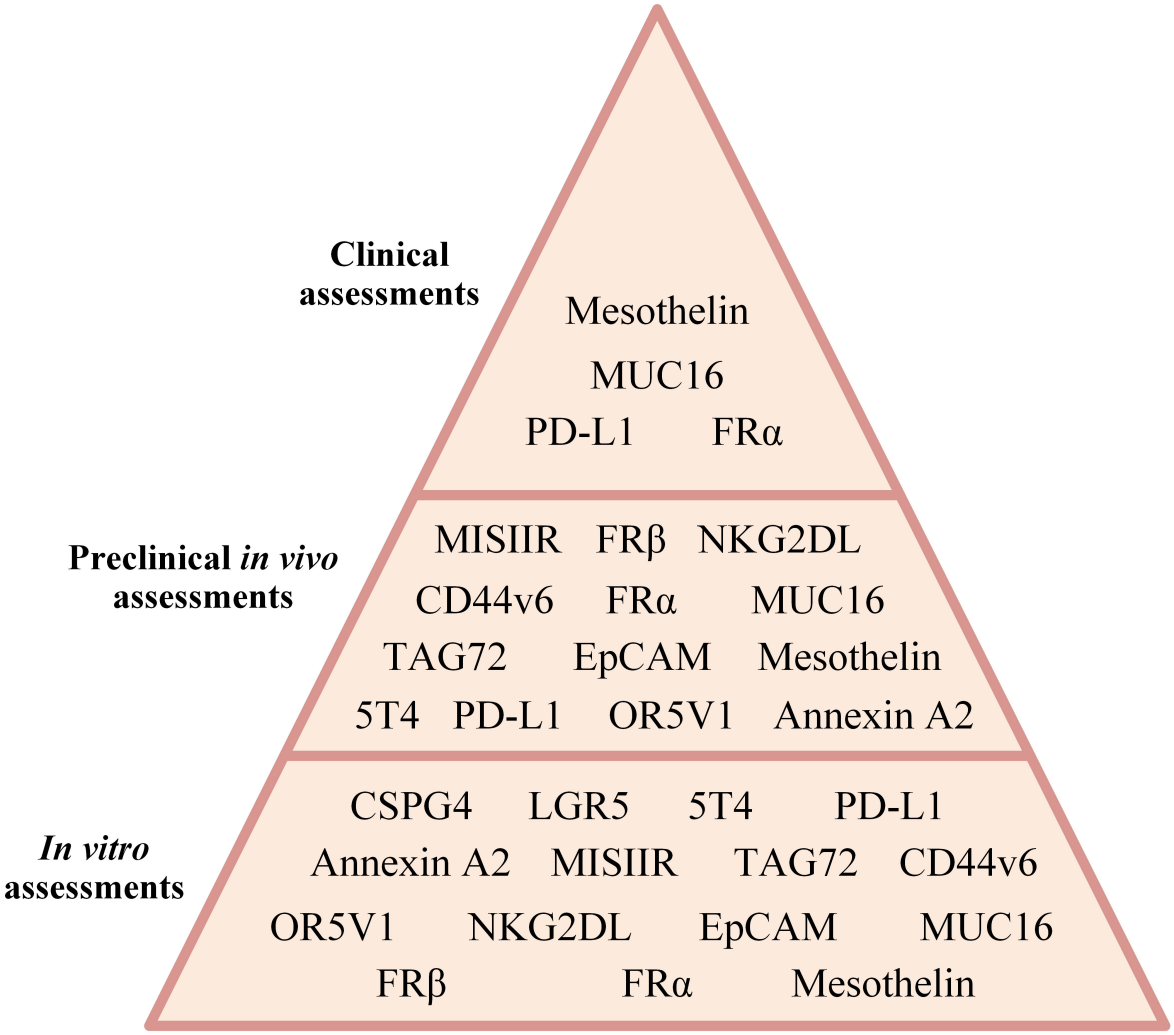
A summary of CAR-T therapy of different ovarian cancer-associated antigens assessed in different investigational stages.

CAR-Ts are T lymphocytes modified to surface-display CARs by the means of which their cytotoxicity is redirected against cells proficient in the expression of tumor-associated antigens (TAAs) or tumor-specific antigens (TSAs) (20). To date, numerous research teams around the world have put an enormous amount of effort into the assessment of CAR-T for the treatment of a wide range of hematologic cancers (including B-cell leukemias and lymphomas, MM, as well as T-cell neoplasms) and solid tumors (including, gliomas, gastrointestinal cancers, thyroid cancer, prostate cancer, breast cancer, lung cancer, cervical cancer, head and neck cancer, and ovarian cancer). Among the targeted antigens, CD19, BCMA, CD22, CD20, CD30, CD123, HER2, EGFR, VEGF-R, Claudin, PD-L1, B7-H3, GD2 TROP-2, MUC1, MUC16, CGRP, CD38, SLAMF7, EpCAM, etc., are the most investigated ones. Recently, researchers have developed CAR-T products and applied them to ovarian cancer for therapeutic purposes. In this article, we comprehensively and in a detailed manner review CAR-Ts developed and investigated for the treatment of ovarian cancer in different experimental stages (focusing on different ovarian cancer cell lines, various cell line- or patient-derived xenograft-based preclinical animal models, or individuals with different stages of ovarian cancer) (**Figure 3**). We will also detail how researchers develop counterstrategies to overcome various limitations of CAR-T therapy in ovarian cancer, including poor CAR-T trafficking and persistence, as well as low antigen density of tumor cells.

## 2 Immunotherapy in ovarian cancer

The advent of targeted immunotherapy has been considered a silver lining in the treatment of cancer patients. With fewer adverse events and higher remission rates, this treatment modality is slowly moving to the front line of treatments for patients with cancer. Among different types of immunotherapies, mAb-based and T-cell-based therapies are considered the most clinically efficient ones. In the past decade, mAb-based cancer treatment modalities have been a hot topic for the treatment of both hematologic malignancies and solid tumors. Since the FDA approval of rituximab for the treatment of patients with chemotherapy regimen-resistant B-cell non-Hodgkin lymphomas in 1997, many other mAbs have also been granted permission for medical use in the field of cancer treatment (21). mAb-based therapies have proven efficient for the treatment of a wide range of solid tumors in which other types of immune-based therapies may provide minimal clinical benefit. This is why mAbs are considered a cornerstone in the treatment of many types of solid tumors including ovarian cancer. In this regard, various antigens have been investigated in the field of mAb-based therapy for ovarian cancer (**Table 1**). Among these target antigens, VEGF, EGFR, EpCAM, FRα, CA125, MUC1, PD-1, PD-L1, and CTLA-4 are the ones comprehensively investigated. Alongside mAb-based therapies, other types of novel immunotherapeutic approaches are being investigated against ovarian cancer, which including T-cell-based therapies. T-cell receptors (TCRs) are antigen receptors expressed on the surface of T cells. α/β T cells are a group of T lymphocytes in which the TCR is composed of an α and a β chain that function together and recognize antigenic peptide fragments that are presented by major histocompatibility complexes (MHCs) (22). T cells have been used for the aim of cancer therapy in different methods including TILs, TCR-engineered T cells, and CAR-Ts (23, 24). TCR-engineered T cells are T lymphocytes that have been genetically engineered to recognize a specific peptide antigen presented by a particular type of MHC. This process entails the genetic engineering of T cells for the expression of TCR α and β chains which are specific for a certain antigen. These α and β chains come from the genes of an activated T cell (24). Using this method, TCR-engineered T cells demonstrate enhanced affinity and specificity to desired cancer antigens. Dissimilar to CAR-Ts which are only capable of interacting with cell surface-expressed antigens, TCR-engineered T cells can interact with MHC-presented intracellular and surface proteins (24). TCR-engineered T-cell therapy has been investigated in a wide range of malignancies including ovarian cancer. Wilms’ tumor protein 1 (WT1), melanoma-associated antigen 4 (MAGE-A4), and New York esophageal-1 (NY-ESO-1) are among the targets investigated in this regard. For instance, Kyi et al. reported the results Phase I clinical trial (NCT00562640) investigating the safety and feasibility of autologous WT1-specific T lymphocytes in patients with recurrent ovarian cancer (25). The enrolled patients included twelve patients aged between 23 to 72 years old and who had at least 4 lines of prior failed therapies. According to the results, no dose-limiting toxicities (DLTs) were documented during the course of the study. Median PFS and median OS were reported as 1.8 and 11.0 months, respectively. Moreover, 1-year PFS and 1-year OS were reported to be 8.3% and 41.7%, respectively. Of note, stable disease was only observed in one patient; whereas eleven others experienced progressive disease (25). It was reported that there was a rise in the level of WT1-specific cytotoxic T lymphocyte precursors in 9 out of 12 patients after the treatment course. Overall, such studies can conclude that TCR-engineered T cells with antigen-specific TCRs are well-tolerated in patients and can mediate mild therapeutic benefits in patients with recurrent ovarian cancer (25). Of note, more clinical trials with broader patient populations are required to elucidate the safety and clinical efficacy of TCR-engineered T-cell therapy in ovarian cancer.

**Table 1:** Monoclonal antibodies FDA-approved or under investigation for the treatment of patients with ovarian cancer (from 2015 to August 2023).

Generic name	Trade name/investigational name	Target	Format	Notes
**Bevacizumab**	Avastin^®^	VEGF	Whole antibody	FDA-approved in 2018 for ovarian cancer
**Mirvetuximab soravtansine**	Elahere^®^	FR-α	Whole antibody conjugated to a drug	FDA-approved in 2022 for ovarian cancer
**Catumaxomab**	Removab^®^	EpCAM/CD3	Trifunctional antibody	NCT00822809
**Cetuximab**	Erbitux^®^	EGF receptor	Whole antibody	NCT00086892
**Panitumumab**	Vectibix^®^	EGFR	Whole antibody	NCT01388621
**Farletuzumab**	MORAb-003	FR-α	Whole antibody	NCT00849667
**Oregovomab**	OvaRex^®^ MAb-B43.13	CA125	Whole antibody	NCT04498117
**Amatuximab**	MORAb-009	Mesothelin	Whole antibody	NCT00325494
**Atezolizumab**	Tecentriq^®^	PD-L1	Whole antibody	NCT03038100
**Tisotumab vedotin**	Tivdak^®^	Tissue factor (TF)	Whole antibody conjugated to a drug	NCT03657043
**Durvalumab**	Imfinzi^®^	PD-L1	Whole antibody	NCT04742075, NCT03899610
**Nivolumab**	Opdivo^®^	PD-1	Whole antibody	NCT05601752
**Pembrolizumab**	Keytruda^®^	PD-1	Whole antibody	NCT02674061, NCT02865811
**Ipilimumab**	Yervoy^®^	CTLA-4	Whole antibody	NCT02498600
**Sabatolimab**	MBG453	Mucin domain-3 (TIM-3)	Whole antibody	NCT02608268
**Spartalizumab**	PDR001	PD-1	Whole antibody	NCT02608268
**Avelumab**	Bavencio^®^	PD-L1	Whole antibody	NCT02580058
**Magrolimab**	Hu5F9-G4	CD47	Whole antibody	NCT03558139, NCT02216409
**-**	hu3S193	Lewis-Y	–	NCT01137071, NCT00617773
**Anetumab ravtansine**	BAY 94–9343	Mesothelin	Whole antibody conjugated to a drug	NCT02751918
**Navicixizumab**	–	Vascular endothelial growth factor (VEGF) and delta-like ligand 4 (DDL4)	Bispecific	NCT03030287
**Lifastuzumab Vedotin**	LIFA	NaPi2b	Whole antibody conjugated to a drug	NCT01911598
**-**	TQB2450	PD-L1	Whole antibody	NCT04236362
**-**	INCAGN01949	OX40	Whole antibody	NCT02923349
**-**	DMUC5754A	MUC16	Whole antibody conjugated to a drug	NCT01335958
**Abagovomab**	–	CA125	Whole antibody	NCT00418574
**Tocilizumab**	Actemra^®^	IL-6 receptor	Whole antibody	NCT01637532
**Trastuzumab**	Herceptin^®^	HER2/neu	Whole antibody	NCT00189579
**Ganitumab**	AMG 479	Type 1 insulin-like growth factor receptor (IGF-1R)	Whole antibody	NCT00719212
**Tremelimumab**	Imjudo^®^	CTLA-4	Whole antibody	NCT03899610
**Olaratumab**	Lartruvo^®^	Platelet-derived growth factor receptor-α (PDGFR-α)	Whole antibody	NCT00913835
**-**	DMUC4064A	MUC16	Whole antibody conjugated to a drug	NCT02146313
**Pertuzumab**	Perjeta^®^	HER2	Whole antibody	NCT01684878
**Camrelizumab**	SHR-1210	PD1	Whole antibody	NCT03827837
**-**	RO5323441	Placental growth factor (PlGF)	Whole antibody	NCT01148758
**Dostarlimab**	Jemperli^®^	PDCD1	Whole antibody	NCT04679064
**-**	HuMax-IL8	IL-8	Whole antibody	NCT02536469
**-**	MOv18	FR-α	Whole antibody	NCT02546921
**Gatipotuzumab**	PankoMab-GEX	TA-MUC1	Whole antibody	NCT01899599, NCT01222624
**-**	LY3022855	CSF-1R	Whole antibody	NCT02718911
**Enoticumab**	REGN421	DDL4	Whole antibody	NCT00871559
**Seribantumab**	MM-121	ErbB3	Whole antibody	NCT01447706
**-**	DKN-01	DKK1	Whole antibody	NCT03395080
**Monalizumab**	IPH2201	CD94/NKG2	Whole antibody	NCT02671435
**Conatumumab**	AMG-655	TRAIL-R2 (CD262)	Whole antibody	NCT00819169
**-**	PF-06647263	EFNA4	Whole antibody conjugated to a drug	NCT02078752
	IMGN901	CD56	Whole antibody	NCT00346385
**Telisotuzumab**	ABT-700	c-Met	Whole antibody	NCT01472016
**Figitumumab**	CP-751871	IGF-1 receptor	Whole antibody	–
**Imalumab**	BAX69	Macrophage inhibitory factor (MIF)	Whole antibody	NCT01765790

**Figure 4:**
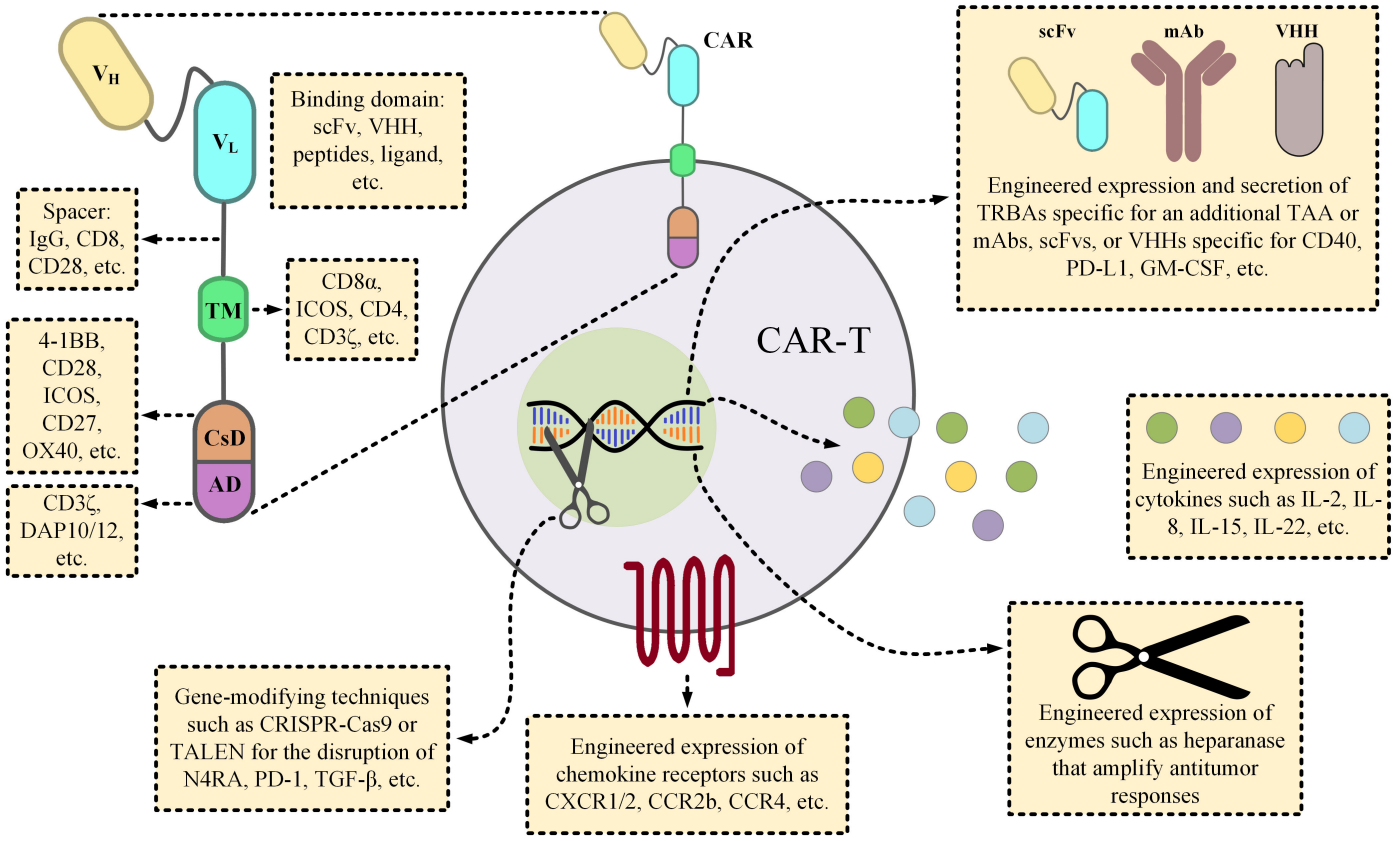
The details of a CAR molecule and the various strategic twists proposed by scientists to overcome the obstacles of CAR-T therapies. From the early days of CAR-T development, various components have been incorporated into CAR constructs. Since such components could substantially influence the functionality of the CAR-Ts, their selection has been a subject of paramount importance. For instance, the length of the spacer fragment can influence the cytokine production profile of the CAR-Ts, or the choice of the co-stimulatory domain(s) can have substantial impacts on the expansion, persistence, or even the phenotypic features of the CAR-Ts. Researchers have engineered CAR-Ts to secrete TRBAs (for example against a different TAA, rather than the one targeted by CAR-Ts, to augment antitumor responses) or other forms of mAbs for neutralizing the mediators of cytokine release syndrome (CRS) or to counteract immunosuppression. CAR-Ts can also be manipulated to produce factors, such as ILs, that boost their effector function, or to express enzymes that help modify the components of the tumor microenvironment for facilitating intratumoral trafficking of the immune effector cells. In regard to increasing CAR-T accumulation in tumor sites, investigators have also engineered CAR-Ts to express the cognate receptors of the molecules that are overexpressed by tumor cells, and they have reported encouraging results (27, 28). Ultimately, CAR-T therapies can also benefit from the novel gene-modifying techniques in a way that they can be utilized for the generation of immunosuppression-resistant or *off-the-shelf* CAR-Ts through the disruption of immunosuppressive genes or TCR αβ chains, respectively. AD, activation domain; CsD, costimulatory domain; GM-CSF, granulocyte-macrophage colony-stimulating factor; ICOS, inducible T-cell costimulator; IL, interleukin; mAb, monoclonal antibody; scFv, single-chain variable fragment; TAA, tumor-associated antigen; TALEN, transcription activator-like effector nucleases; TCR, T-cell receptor; TM, transmembrane domain; TRBA, T-cell-redirecting bispecific antibodies.

CAR-Ts are T cells genetically engineered to express CARs on their surface by the means of which they are capable of targeting cells proficient in the expression of TAAs or TSAs of interest in a fashion similar to that of mAbs and without the engagement of MHC. The genetic manipulation of CAR-Ts usually entails the application of viral gene introduction techniques (such as by the means of lentiviral or retroviral particles) or non-viral gene introduction methods (including transposons, mRNA electroporation, etc.) (26). In reference to the structural characteristics of CAR molecules, CARs are composed of three topological domains; extracellular domain, transmembrane domain, and intracellular domains (**Figure 4**). The extracellular domain harbors the antigen-recognition domain and a fragment that bridges this domain to the transmembrane domain, known as the spacer. Researchers have used single-chain variable fragments (scFvs) or single domains on a heavy chain (VHH) as the antigen-recognition moieties of CARs; however, other applicable fragments have also been incorporated into CARs for this aim (29). The signaling domains that initiate the downstream signaling cascades of CAR-Ts necessary for efficient activation and antitumor responses are located at the intracellular portion of these chimeric receptors. Depending on the generation of the CAR construct, CARs could have no, one, and two co-stimulatory domains (1^st^-, 2^nd^-, and 3^rd^-generation CARs, respectively). Other generations of CARs have also been devised, and they are structurally tailored versions of the 2^nd^-generation CARs as 4^th^- and 5^th^-generation CARs are endowed with an inducer for the production and secretion of cytokines of interest or the intracellular receptor fragment that responds to cytokine stimuli, respectively. Over the past years, researchers have further engineered different platforms of CAR-Ts, each of which developed to be capable of overcoming certain limitations entwined with this treatment modality (**Figure 4**).

CAR-Ts mediate cytolytic reactions (by means of secreting perforin and granzyme B, as well as various proinflammatory cytokines) against target cells upon the engagement of their chimeric receptors with the indicated target antigen(s). CAR-T products are conventionally administered through the intravenous route. In the context of blood-based oncological indications, these engineered cells can easily encounter the cells proficient in the expression of the indicated target antigen, and efficiently eliminate them (30). This phenomenon has rendered CAR-T therapy an applicable frontline treatment for certain patients with hematologic cancers. However, CAR-T therapy has encountered serious limitations in the context of solid tumors and researchers have put tremendous effort into addressing these hindrances in recent years (20, 30). Lack of qualified target antigens, the immunosuppression and metabolic shortcomings of the tumor microenvironment (TME) impinging on the infused T cells, and ineffective trafficking into tumor sites are all reasons for the failure of CAR-T therapy in the treatment of solid tumors (20, 30).

## 3 CAR-T therapy obstacles in solid tumors

At first, CAR-T therapy was proposed as a novel treatment modality against solid tumors; however, this novel treatment managed to mediate more prominent remissions in patients with certain blood-based malignancies over the course of the past years. Such unexpected outcomes accentuated the bold differences that exist between the CAR-T therapy of solid tumors and hematologic malignancies (20, 30). Researchers have proposed and evaluated various counterstrategies for bypassing these hindrances; however, tremendous effort and time need to be put into better identifying the behavior and characteristics of solid tumors beforehand. Some of these differences are briefly outlined in this section.

### 3.1 Tumor accessibility

CAR-Ts are often administered through the intravenous route. In the bloodstream and lymph nodes, CAR-Ts encounter and consequently engage with their target cells to initiate cytolytic reactions against them (which is basically what happens in the case of lymphomas and leukemias) (31). However, this storyline is not as straight in the context of solid tumors as it is in hematologic cancers. Solid tumor cells tend to aggregate in isolated territories (termed *tumor islets*) that allow them to establish an immunosuppressive environment whilst surrounded by elements that support their immune escape, progression, and invasion (30). Such tumor islets are often populated with tumor-associated blood vessels that are morphologically different from the healthy tissue vessels (30). The endothelial cells of these abnormal vessels usually undergo modifications (induced by tumor-secreted factors) that result in impaired expression of molecules (such as VCAM-1, ICAM-1, ICAM-2, etc.) that correspond to those expressed by T cells and necessary for extravasation (32, 33). Moreover, pericytes offer structural integrity to the mentioned blood vessels which leads to further tumor progression (34). Another important role in this scenario is played by cancer-associated fibroblasts (CAFs) (35, 36). These fibroblasts produce a formidable web of the extracellular matrix (ECM) that remarkably restricts the migration of lymphocytes toward target tumor cells (35, 36). Another factor that limits the accessibility of CAR-Ts to tumor islets is the presence of tumor-associated macrophages (TAMs) as it has been evident that the depletion of these macrophages correlates with an amplified number of T cells in tumor sites (37). Moreover, the metabolic characteristics of the TME could also impinge on the transmigration capacity of CAR-Ts as findings have indicated that non-restricted outgrowth of tumor cells results in hypoxic conditions in the TME, impairing T cell motility (38). Moreover, various distinct signaling pathways also have been known to contribute to the diminished expression of certain chemotactic factors (CCL4, CCL5, etc.) by the cellular elements of the TME which leads to the exclusion of T lymphocytes from tumor islets (39–41).

### 3.2 Immunosuppressive TME

There are various cellular and molecular elements in the TME that act in favor of tumor cells in terms of progression and invasiveness (42, 43). The cellular elements include CAFs, TAMs, Tregs, and myeloid-derived suppressor cells (MDSCs) that impose harsh conditions on T cells restricting or diminishing their antitumor efficacy (42, 43). Moreover, various immunoinhibitory molecules are also secreted and/or surface-displayed by such cellular elements or tumor cells themselves that further impair the functionality of CAR-T/Ts in a pro-tumor fashion within the TME (42, 43).

### 3.3 Tumor heterogeneity

Solid tumor bulks are often populated with different types of malignant cells that exhibit distinct characteristics in terms of behavior (drug resistance patterns) and target antigen expression (43). For instance, in the context of ovarian cancer, certain malignant cells might be proficient in the expression of mesothelin, MISIIR, and EpCAM, whereas there might be tumor cells within a given tumor site that express neither. This occurrence renders it impossible to target all the tumor cells within a tumor site with a single CAR-T product and necessitates precise screening for profiling the antigen expression patterns of the tumor cells prior to targeted immunotherapy. Moreover, the number of antigen molecules displayed over the surface of a tumor cell is an important factor that determines whether a CAR-T can establish productive CAR-antigen engagement with that cell to initiate cytolytic reactions (20). Solid tumor cells might exhibit different rates of expression for a certain target antigen within a TME (20). Low expression rates give tumor cells protection against CAR-T recognition.

### 3.4 Lack of qualified target antigens

The principal concept of CAR-T therapy was developed upon the redirection of modified T cells against malignant cells proficient in the expression of TSAs of interest; however, the practicality of this concept is restricted by a lack of known TSAs. To compensate, researchers considered targeting TAAs. Due to the expression of such TAAs by the cells of healthy tissues, their CAR-T-mediated targeting results in cytotoxic reactions against healthy tissues, which is clinically recognized as “*on-target off-tumor*” effects (43). In the context of CD19-redirected CAR-T products, such unfavorable effects lead to the depletion of normal B cells (regarded as *B cell aplasia*); thereby increasing the risk of bacterial and/or viral infections in the respective patients (31). Such adverse events are manageable through different strategies such as immunoglobulin replacement (31). However, in the context of solid tumors, such unintended effects lead to serious, and sometimes detrimental and irreversible, damage to the vital organs of the recipients (44). Therefore, the research for qualified target antigens in solid tumor CAR-T therapy is a subject of current investigation, and it must continue.

## 4 Target antigens for ovarian cancer CAR-T therapy

### 4.1 Müllerian inhibiting substance type 2 receptor (MISIIR)

The overexpression of the TGF-β family member MISIIR is reported in a high rate of ovarian cancer cases (45). Owing to its scarcity in healthy tissues and its vital role in apoptosis induction in tumors, MISIIR has been considered an interesting target antigen in ovarian cancer immunotherapy, as well as other endometrial malignancies (45). In 2020, Rodriguez-Garcia and colleagues reported the development and assessment of MISIIR-redirected CAR-Ts (using a fully human scFv as the targeting domain of the CAR construct) and reported that these therapeutics mediated MISIIR-dependent cytotoxicity *in vitro* and in preclinical animal models (45). In the *in vitro* experiments, MISIIR-redirected CAR-Ts were capable of mediating cytolytic reactions against a variety of MISIIR-positive cell lines as well as patient-derived cancer cells, while sparing healthy human cells (45). Conclusively, Rodriguez-Garcia and colleagues suggested that MISIIR might be a suitable cancer immunotherapy target antigen for the development of MISIIR-redirected CAR-Ts against MISIIR-positive gynecologic disorders (45). However, broader investigations on the targeting of MISIIR via CAR-Ts are warranted in the context of ovarian cancer.

### 4.2 Olfactory receptor family 5 subfamily V member 1 (OR5V1)

In 2022, Martin and colleagues reported that OR5V1 is expressed in a variety of ovarian cancer histological samples, while its expression in normal tissues is restricted to the testis (46). Owing to these findings, Martin et al. reported the development of an scFv-based 2^nd^-generation CAR-T redirected against the OR5V1 antigen (OR5V1-redirected CAR-Ts) and eventually introduced this construct into human T cells via retroviral transduction (46). The results of the *in vitro* assays demonstrated that OR5V1-redirected CAR-Ts mediated specific tumoricidal effects against HeLa cells in a dose-dependent fashion while managing to spare a panel of normal human cells (46). Moreover, Martin and colleagues established HeLa-bearing preclinical mouse models and reported that the treatment subjects experienced delayed tumor outgrowth under OR5V1-redirected CAR-T treatment (46). Conclusively, these researchers demonstrated the antitumor potential of OR5V1-redirected CAR-T against OR5V1-positive malignancies with negligible toxicities (46). To further elucidate the suitability of OR5V1 as an immunotherapy target antigen, in-depth preclinical and clinical assessments must be taken into consideration.

### 4.3 Annexin A2

In 2018, Cua and colleagues reported the development of a variety of mAbs that target embryonic stem cells of human origin (47). These researchers reported that such mAbs could be applied for or engineered into different mAb-based platforms for therapeutic purposes (47). Among these mAbs, *2448* was reported to recognize a unique antigen on a variety of tumor cells (47). Cua et al. demonstrated that *2448* can potentially bind a specific site on annexin A2, expressed in breast cancer and ovarian cancer (47). Further engineering techniques revealed that *2448* could also mediate antigen-dependent cytotoxicity while applied as an ADC, alongside mediating antibody-dependent cellular cytotoxicity in *in vitro* and animal-based experiments (47). Ultimately, Cua and colleagues suggested that annexin A2 might be taken into consideration as a qualified cancer immunotherapy target antigen and that *2448* could be further applied for therapeutic advantage (47). Annexin A2 has been known to arrange different molecular mechanisms in cancer progression, as its expression fluctuates in different oncological indications (48). In 2020, Leong and colleagues derived a CAR targeting domain from *2448* and developed annexin A2-redirected CAR-Ts (49). In detail, Leong et al. evaluated the effects of different CAR spacer domains (short, intermediate, and long; with 12, 122, 229 amino acids, respectively) on the cytotoxic activity and proinflammatory cytokine secretion ability of the developed annexin A2-redirected CAR-Ts, and reported that CAR-Ts with the longer spacer fragment outperformed those with the short or intermediate spacer fragments in mediating cytolytic reactions against the annexin A2-positive cell line IGROV-1 (49). Moreover, it was demonstrated that the tumoricidal effects mediated by the annexin A2-redirected CAR-Ts (with the long spacer) were annexin A2-dependent, as they were only against the IGROV-1 and SKOV-3 cell lines, but not the IMR90 and HFF-1 cell lines (49). Leong et al. also reported more encouraging results as their annexin A2-redirected CAR-Ts prolonged the survival of ovarian cancer xenograft animals and shrunk tumors by almost 75% (49). Such novel findings accentuate the fact that annexin A2 might be considered a therapeutic target antigen for ovarian cancer, as well as breast cancer; however, broader preclinical and substantiated clinical evidence is required before confidently classifying this antigen as a potential target.

### 4.4 Chondroitin sulfate proteoglycan 4 (CSPG4)

The expression of CSPG4 has been reported in different oncological indications, including ovarian cancer, breast cancer, glioblastoma, etc., which renders this antigen as an interesting target antigen in targeted immunotherapy (50). This antigen can also be leveraged for targeting tumor-associated vasculature, owing to its expression on the blood vessel cells of malignant tissues (51). In recent years, researchers have attributed a number of roles to CSPG4, which might further validate its importance as a target antigen; such roles include contribution to the formation of metastatic lesions, contribution to tumor aggressiveness, and arranging tumor progression signals (52, 53). Based on these findings that implicate the importance of CSPG4 for tumors proficient in its expression, researchers have proposed that it is very unlikely for tumor cells to undertake mechanisms for CSPG4 down-regulation and/or loss following its targeting via CAR-Ts (54). In 2022, Harrer et al. reported the results of a study investigating the drug-induced expression of CSPG4 on the SKOV-3 ovarian cancer cell line, and targeting these cells using CSPG-4-redirected CAR-Ts (54). In detail, these researchers used decitabine (5-aza-2-deoxycytidine), a US FDA-approved medication for the treatment of myelodysplastic syndrome (MS), to induce the expression of CSPG4 on the surface of SKOV-3 cells (54). Decitabine is a DNA methylation inhibitor that has been known to have upregulatory effects on the expression level of CSPG4 in a number of melanoma cell lines (55). Generally, decitabine and its structural relatives such as azacitidine have been reported to induce unregulated expression of various TAAs on cancer cells, rendering CAR-T-mediated targeting of these target antigens more feasible (56). First, these researchers demonstrated that decitabine mediates CSPG4 upregulation in SKOV-3 cells in a dose-dependent manner (54). Of note, SKOV-3 cells are deficient in the expression of CSPG4 (57). Next, Harrer and colleagues co-cultivated CSPG4-positive SKOV-3 cells with mRNA-electroporated 2^nd^-generation CSPG4-redirected CAR-Ts (54). The results indicated that CSPG4-redirected CAR-Ts mediated effective target antigen-specific antitumor activity against ovarian cancer cells induced to express CSPG4, in four different effector-to-target ratios (54). Additionally, CSPG4-redirected CAR-Ts showed remarkable IFN-γ production and secretion following being subjected to decitabine-treated CSPG4-expressing SKOV-3 cells (54). However, it is worth mentioning that CSPG4 is not generally expressed by ovarian cancer cells; therefore, it cannot be considered a target antigen for CAR-T therapy of ovarian cancer, but the results of this study can highlight the potential of this cell surface antigen as a secondary inducible target antigen for CAR-T therapy of various types of solid tumors including ovarian cancer. Moreover, the effects of decitabine for inducing CSGP4 expression in ovarian cancer cells should be more broadly investigated since the ovarian cancer cells used in the mentioned study are not patient-derived primary samples. Also, preclinical data using mouse models are critically required for evaluating the safety and efficacy of the methods described above. Above all this, CSPG4 has been employed as a CAR-T therapy target in various types of malignancies including glioblastoma and B-cell precursor leukemia, which highlights the importance of this target antigen for different types of immunotherapies (58–60).

### 4.5 Leucine-rich repeat-containing G protein-coupled receptor 5 (LGR5)

Wang and colleagues have reported the expression of LGR5 in a variety of ovarian cancer cell lines (including OAW28, COV318, and COV362), and have also reported its elevated expression level in ovarian cancer tissue samples in patients who had relapsed tumors (61). It has been demonstrated that this antigen has distinctive roles in cancer emergence and metastasis, and its expression has also been reported in a variety of malignancies including colon cancer and ovarian cancer (62). In this regard, Wang et al. generated LGR5-redirected CAR-Ts and set out to evaluate their tumoricidal effects on different cell lines of ovarian cancer origin and patient-derived tumor cells in monolayer and 3D culture conditions (61). In the monolayer model, Wang et al. reported that their LGR5-redirected CAR-Ts mediated tumoricidal effects in an antigen density-dependent fashion, as they remarkably suppressed the outgrowth of the ovarian cancer cell lines COV318 and COV362 (which overexpress LGR5), while not enforcing the same reactions against SKOV3 and OV90 cell lines that display LGR5 on their surface at lower levels (61). Moreover, LGR5-redirected CAR-Ts also mediated pronounced cytolytic reactions against patient-derived ovarian cancer tumor cells (61). In reference to the 3D culture condition experiments, LGR5-redirected CAR-Ts mediated tumoricidal effects against patient-derived ovarian tumor cells and the OAW28, COV318, and COV362 cell lines (61). According to another investigation, Thompson and colleagues developed a real-time cytotoxicity assay that could be applied in laboratory settings to efficiently compare the tumoricidal effects of different CAR-T platforms (to reach a candidate therapeutic for future clinical assessments) (62). Using this technique, Thompson et al. were able to establish a measure (which was defined as the time CAR-Ts needed to enforce tumoricidal reactions against 50% of the target tumor cells) to opt for a certain LGR5-redirected CAR-T product as their therapeutic candidate for clinical assessment (called *CNA3103*) (62). Moreover, these researchers reported that their therapeutic candidate was able to remarkably exhibit tumoricidal reactions against different cell lines of ovarian cancer and colon cancer origin (62). Ultimately, Thompson et al. suggested that this technique might also be applied in Phase I dose-escalation clinical trials for dose optimization purposes (62). Aside from the expression of LGR5 in ovarian cancer, its expression in colorectal cancer might also support its suitability as a target antigen in the CAR-T therapy of colorectal cancer (63). According to a 2022 report by McPeake and colleagues, LGR5-redirected CAR-Ts might be promising therapeutics for the treatment of colorectal cancer as administration of *CNA3103* into preclinical mouse models mediated complete remission in all of the subjects (100%) (63). Moreover, these researchers also reported that IV administration of *CNA3103* (5 × 10^6^) into mouse models mediated remission and prolonged their survival remarkably (63). According to a recent report, Bandara and colleagues developed a panel of six different LGR5-redirected CAR-T products and demonstrated that four of these products were capable of mediating meaningful tumoricidal effects *in vitro* against cell lines of colorectal cancer (64). Furthermore, the investigators also evaluated these four products in xenograft models of human colorectal cancer and reported that three CAR-T products mediated significant tumor suppression (64). Such studies might highlight the potential suitability of LGR5 as a target antigen for the CAR-T therapy of ovarian cancer, as well as colorectal cancer; however, such CAR-T products need to be thoroughly and strictly evaluated in clinical settings to substantiate such claims.

### 4.6 CD44v6

CD44v6 has been recognized as a variant of CD44 whose role in tumor proliferation, aggressiveness, and migration in a variety of malignancies (including breast cancer, ovarian cancer, head and neck epithelia, colorectal cancer, etc.) has been evident, according to experimental findings (65–68). Based on the findings achieved in preclinical experiments, targeting CD44v6 has been correlated with tumor outgrowth suppression in multiple myeloma (MM) and acute myeloid leukemia (AML) (65). Recently, Porcellini and colleagues developed CD44v6-redirected CAR-Ts, which were also equipped with an at-will depletion switch, and assessed their antitumor efficacy against CD44v6-positive cell lines (IGROV-1 of ovarian cancer origin and MR232 of lung cancer origin) and in preclinical mouse models (65). Porcellini et al. reported that their CD44v6-redirected CAR-Ts mainly expressed the markers of T memory stem cells (T_scm_) and T central memory cells (T_cm_) subsets, and they exhibited CD44v6-dependent expansion and tumoricidal effects upon co-cultivation with the MR232 and IGROV-1 cell lines (65). Moreover, the researchers established IGROV-1-bearing immunodeficient NSG mouse models and reported that CD44v6-redirected CAR-Ts infiltrated and proliferated at tumor foci upon intravenous administration, which eventually culminated in meaningful tumor outgrowth suppression (65). Conclusively, Porcellini and colleagues asserted that CD44v6-redirected CAR-Ts could be of therapeutic advantage for the treatment of CD44v6-positive ovarian cancer, as well as other related malignancies; however, carefully conducted clinical evaluations are to be taken into consideration before drawing such conclusions (65). Other researchers have also evaluated the antitumor efficacy of CD44v6-redirected CAR-Ts in the context of other malignancies (69). For instance, Haist and colleagues developed CD44v6-redirected CAR-Ts and demonstrated a direct pattern between the expression level of CD44v6 by primary blasts of human head and neck squamous cell carcinoma and the tumoricidal effects of CD44v6-redirected CAR-Ts (69). In the context of AML, Tang and colleagues reported that individuals with AML, or SKM-1 and K562 cell lines, with the FLT3 or DNMT3A mutations had higher expression levels of CD44v6 (in comparison with the patients without the mentioned mutations) (70). Furthermore, these researchers developed CD44v6-redirected CAR-Ts and demonstrated that these effector cells secreted proinflammatory cytokines and exhibited CD44v6-dependent tumoricidal effects upon co-cultivation with CD44v6-positive cells (while sparing CD44v6-negative cells), which were also consistent with the outcomes of their *in vivo* experiments (70). Ultimately, Tang and colleagues asserted that CAR-T-mediated targeting of CD44v6 in patients with FLT3 or DNMT3A mutations might serve as a qualified therapeutic option; however, clinical scrutiny is warranted in this matter (70). According to another study, Casucci and colleagues also generated CD44v6-redirected CAR-Ts and demonstrated that these effector cells were capable of enforcing tumoricidal effects against primary blasts of AML and MM patients while managing not to attack hematopoietic stem cells (71). Recently, Stornaiuolo and colleagues published the results of an investigation that aimed to optimize the tumoricidal efficacy of CD44v6-redirected CAR-Ts by focusing on the structure of the CAR spacer domain, which was derived from a fragment of the human low-affinity nerve growth factor receptor (LNGFR) (72). The investigators attributed the varied phenotypes of the generated CAR-Ts to the length of the different spacer fragments incorporated into the CAR constructs of each of the developed CAR-T products, which might in their own way affect the level of spontaneous antigen-independent signaling and the rate of CAR surface presentation (72). The researchers reported that one of the CAR-T products (designated as CD44v6-NWN2.CAR-Ts) exhibited pronounced tumoricidal effects *in vitro* and *in vivo*, whose T cell populations were mainly composed of T_cm_ (72). Such investigations accentuate the importance of how CAR design might remarkably affect the antitumor efficacy of the developed CAR-Ts (60, 72).

### 4.7 PD-L1

Peritoneal metastasis of ovarian tumors has often been reported in patients with ovarian tumors, and these malignant cells somehow manage to escape immunosurveillance; however, the exact underlying mechanism for this occurrence is not completely deciphered (73). To this aim, Abiko and colleagues conducted an investigation focused on assessing the correlation between PD-L1 expression and tumor metastasis to the peritoneum (73). According to the findings, these researchers reported a direct relationship between PD-L1 expression by human ovarian tumors and metastasis to the peritoneum, as achieved through immunohistochemistry and microarray techniques (73). Moreover, it was reported that the overexpression of PD-L1 had debilitating impacts on the cytolytic reactions of cytotoxic T lymphocytes, as well as their degranulation, whereas PD-L1 deficiency improved the antitumor effects exerted by such lymphocytes (73). Furthermore, these researchers demonstrated that cytotoxic T lymphocytes exhibited an exhausted gene expression profile which was attributed to PD-L1 overexpression by ovarian tumors (73). Abiko et al. also conducted preclinical experiments and reported that PD-L1 deficiency culminated in suppressed peritoneal tumor outgrowth correlating with protracted survival in the animal subjects (73). Ultimately, these investigators asserted that PD-L1 plays an important role in promoting tumor metastasis to the peritoneum, as achieved through exhausting functional cytotoxic T lymphocytes, and they also proposed that targeted therapy of this antigen is a potential strategy for preventing this occurrence (73). Such studies accentuate the importance of PD-L1 in solid tumors, especially ovarian cancer. In 2022, Ma and colleagues reported that the tumoricidal efficacy of 2^nd^ generation HER2-redirected CAR-Ts was hampered by the component of malignant pleural effusion (MPE) or malignant ascites (MA), leading to a compromised expansion and cytokine production ability of the CAR-Ts (74). These researchers investigated the reason for this occurrence and identified a high-level expression of PD-L1 by the cells of MPE/MA for this negative impact (74). Of note, MPE/MA is often reported in individuals with advanced non-hematologic malignancies, such as ovarian cancer, which also coincides with tumor metastasis to the peritoneum (74). Such characteristics have been identified as potential elements that hamper the therapeutic effects of CAR-T treatments, through the formation of a highly immunosuppressive TME (74). To overcome this limitation, Ma and colleagues developed CAR-Ts engineered to co-express two distinct CAR constructs; one of them was a 2^nd^ generation chimeric receptor redirected against HER2, whereas the other one was composed of a PD-L1-specific scFv fused to the 4-1BB co-stimulatory domain (referred to as PD-L1.BB CSR) (74). It was reported that co-expression and subsequent engagement of the PD-L1-specific CAR molecules on the surface of the HER2-redirected CAR-Ts enabled them to outperform conventional CAR-Ts in terms of expansion rate and counteract the suppressive effects of MPE/MA upon their co-cultivation with irradiated SKOV3 cell line (proficient in the expression of PD-L1) (74). Moreover, Ma and colleagues further evaluated the efficacy of PD-L1.BB CSR-positive HER2-redirected CAR-Ts in mediating prolonged survival in xenograft models of pleural and peritoneal metastasis (74). Briefly, the investigators established pleural and peritoneal metastasis NSG mouse models (using the PD-L1-positive cell line SKOV3, or the lung adenocarcinoma cell line A549), ten days following which, the animal models underwent CAR-T treatment via the intrapleural or intraperitoneal route (74). According to the results, PD-L1.BB CSR-positive HER2-redirected CAR-Ts were able to mediate prolonged elimination of the established tumors (74). The investigators asserted that both components of the PD-L1-specific chimeric receptor (scFv and the 4-1BB signaling domain) were responsible for the enhanced efficacy of the CAR-Ts (74). To further investigate this concept, in March 2021, a clinical investigation was initiated with eighteen individuals diagnosed with HER2-positive malignancies with pleural or peritoneal metastatic tumors. No findings have yet been published.

### 4.8 5T4

5T4 is a 72 kDa oncofetal antigen (which is also recognized as trophoblast glycoprotein) that might be considered a qualified cancer immunotherapy target antigen as it exhibits a restricted expression profile in healthy cells, but its expression is remarkably elevated in different stages of cancer progression of different types of tumors, including ovarian cancer (75). In 2018, Owens et al. generated two distinct sets of CAR-Ts redirected against 5T4 using two different mAb derivatives (named *H8* and *2E4*) and evaluated the antitumor activity of these effector cells both *in vitro* and *in vivo* (76). Of note, it was reported that these two mAbs exhibited different affinities for their target antigen, 5T4 (76). Briefly, *in vitro* assessments of these researchers included co-cultivation of 5T4-redirected CAR-Ts with autologous cancer cells as well as two ovarian cancer cell lines, including SKOV-3 and OVCAR-3 (76). Both 5T4-redirected CAR-T products produced high levels of INF-γ upon encountering the ovarian cancer cell lines and autologous cell samples (76). Moreover, these CAR-Ts also managed to produce and secrete moderate and minimum levels of IL-2 upon co-cultivation with the ovarian cancer cell lines and autologous tumor cells, respectively (76). In addition to these outcomes, Owens et al. used preclinical mouse models established using luciferase-expressing SKOV-3 cells (76). According to the results, 5T4-redirected CAR-T treatment culminated in the therapeutic benefit of the preclinical animals against the established tumors (76). Ultimately, Owens and colleagues asserted that the isolation of T cells from ovarian cancer patients and their reprogramming for the expression of 5T4-redirected CAR molecules can lead to the targeted responses of the engineered T cells to autologous cancer cells proficient in the expression of the targeted antigen (76). Recently, Guo and colleagues developed 2^nd^-generation 5T4-redirected CAR-Ts and aimed to assess their antitumor functionality and proinflammatory cytokine secretion ability upon their co-cultivation with a variety of 5T4-positive ovarian cancer cell lines, including SKOV3, A2780, and ES2 cell (76). These researchers reported that their 5T4-redirected CAR-Ts mediated remarkable tumoricidal effects against the mentioned cell lines *in vitro*, alongside secreting high levels of GM-CSF, IFN-γ, and IL-2 (76). Moreover, Guo et al. also established xenograft models of peritoneal ovarian cancer by infusing SKOV3-luc cells into B-NDG mouse models (76). Following localized administration of the 5T4-redirected CAR-Ts into the animal subjects, it was reported that the treatment managed to hinder tumor outgrowth; therefore, prolonging the survival of the animal subjects (76). Ultimately, the investigators suggested that this study provided a groundwork for the future clinical assessment of 5T4-redirected CAR-Ts in 5T4-positive ovarian cancer patients (76). Other researchers have also assessed the antitumor efficacy of T cell-based immunotherapies in other 5T4-positive malignancies, including renal cell carcinoma (RCC) (77). For instance, an investigational team previously reported that they identified high-avidity T-cell clones that could specifically target tumor cells that display a certain epitope of 5T4 (amino acids 17 to 25) via HLA-A2 (77). Briefly, single-cell RNA sequencing was employed to identify the TCRα and TCRβ sequences of these T cell clones, and eventually whole TCRα and TCRβ sequences were introduced into T cells via lentiviral transduction (77). Of note, seven different pairs of TCRα/β were identified over the course of this study (77). The results of the *in vitro* experiments demonstrated that CD8+ TCR-engineered T cells secreted proinflammatory cytokines and mediated targeted tumoricidal effects upon encountering target cells (solid tumor cell lines and primary RCC cells) displaying the mentioned epitope of 5T4 (77). Moreover, the researchers also reported that the engineered T cells were capable of recognizing the surface-presented peptide of 5T4 in T2 cells (77). Ultimately, Xu and colleagues suggested that T cells engineered with novel TCRs capable of recognizing the mentioned 5T4 epitope might be of therapeutic advantage for the treatment of 5T4-positive individuals with HLA-A2-positive indications; however, following careful and sufficient clinical examinations (77).

### 4.9 TAG72

Investigators have proven that abnormally glycosylated forms of target antigens could be of interest in targeted cancer immunotherapy (20, 43). Among such glycoforms, TAG72 exhibits an elevated expression profile in various oncological indications, including ovarian cancer (20, 43). In detail, TAG72 is a sialyl Tn hapten found in various glycosylated proteins (20). In 2018, Murad and colleagues incorporated a humanized TAG72-specific targeting fragment into the construct of a 4-1BB/CD3ζ-based CAR and developed TAG72-redirected CAR-Ts (78). The investigators reported that their TAG72-redirected CAR-Ts successfully mediated tumoricidal effects and proinflammatory cytokine secretion in response to TAG72-positive cell lines of ovarian cancer origin (OVCAR3 and OV90) and ascites of corresponding patients (78). The researchers further reported that intraperitoneal administration of TAG72-redirected CAR-Ts into preclinical xenografts of ovarian cancer culminated in suppressed tumor outgrowth and prolonged survival in the treatment subjects, which was more pronounced as repeated administrations were taken into consideration (78). Of note, Murad and colleagues also reported a decline in the expression level of the target antigen, which was parallel with low CAR-T persistence in the treatment subjects (78). Ultimately, the researchers concluded that TAG72-redirected CAR-Ts might be therapeutically valuable for the treatment of TAG72-positive ovarian cancer cases; however, translation of such preclinical findings into clinical assumptions requires in-depth clinical evaluations (78). Other researchers have also assessed the applicability of TAG72-redirected CAR-Ts for the treatment of patients with other solid tumors, such as those with colorectal cancer (79). In a study by Hege and colleagues, the researchers reported the outcomes of two Phase I clinical trials (designated as C-9701 and C-9702) in which metastatic colorectal cancer patients were admitted to undergo first-generation TAG72-redirected CAR-T treatment (79). In the former trial, intravenous CAR-T administration was considered for the subjects (ten patients), whereas in the latter (six patients), metastatic colorectal cancer patients with liver involvement underwent CAR-T administration directly into their hepatic artery (79). According to the results, mild CRS without evident bystander effects against healthy tissues was reported (79). Moreover, the presence of neutralizing antibodies was confirmed in some patients, which were mounted against the humanized targeting domain of the TAG72-redirected CAR-Ts (known as *CC49*) (79). Since the investigators reported no objective tumor responses to the treatment, various points were elucidated over the course of these trials (79). To overcome the issue of anaphylaxis, CAR-Ts could be equipped with fully human or humanized targeting domains, and to tackle the issue of limited CAR-T persistence, the effector cells must be genetically engineered to express CAR molecules that harbor auxiliary stimulatory domains (such as 2^nd^- and 3^rd^-generation CARs) (60, 80). Antigen-dependent tumor escape is one of the most frequently undertaken mechanisms by which tumor cells evade the immune system (30, 42). Targeting more than one antigen might serve as a potential counterstrategy in targeted cancer immunotherapy (60). In the context of ovarian cancer, Shu and colleagues devised a dual CAR-T platform capable of recognizing TAG72 and CD47 on the surface of tumor cells of interest (81). Despite the constitutive expression of CD47 (which acts as a “*don’t eat me*” signal to macrophages) on the surface of a vast variety of tumor cells, it is also displayed on the surface of healthy cells; therefore, to prevent dual CAR-Ts from targeting undesired cells, Shu and colleagues designed a CD47-specific CAR (with an anti-CD47 scFv as the targeting domain) that does not harbor any intracellular signaling domains (81). Upon co-binding of dual CAR-Ts to CD47 and TAG72 on the surface of tumor cells, CAR-Ts could start eradicating TAG72-positive malignant cells (81). Ultimately, Shu and colleagues suggested that this proof-of-concept might also serve as a potential strategy for the treatment of other relatable malignancies (81). To date, a limited number of studies have investigated the safety and tumoricidal efficacy of TAG72-redirected CAR-Ts, which are only in preclinical stages. To further elucidate the suitability of this target antigen and the applicability of TAG72-redirected CAR-Ts for the treatment of ovarian cancer, as well as other TAG72-positive malignancies, profound preclinical and clinical assessments are warranted.

### 4.10 Epithelial cell adhesion molecule (EpCAM)

EpCAM is a membrane-anchored glycosylated protein with physiological expression by epithelial cells and elevated expression levels by malignant cells of various advanced carcinomas (82). This characteristic renders EpCAM a favorable target antigen for the CAR-T therapy of solid tumors, including ovarian cancer. In 2021, Fu et al. generated EpCAM-redirected CAR-Ts and evaluated the antitumor efficacy of these cells *in vitro* and *in vivo* (83). Briefly, these researchers first assessed the expression of EpCAM on ovarian cancer cell lines and patient-derived samples (83). According to the results of the immunohistochemistry analysis, the expression level of EpCAM in ovarian cancer tissue is remarkably higher than its expression in ovarian para-cancerous tissues (83). Moreover, SKOV3 cells exhibited a high level of EpCAM expression (83). In the next step, Fu et al. co-cultured EpCAM-redirected CAR-Ts with SKOV3 cells and evaluated the CAR-T mediated cytotoxicity and the level of INF-γ secretion (83). According to the result of the real-time cell analysis assay, EpCAM-redirected CAR-Ts mediated effective tumoricidal activity against SKOV3 cells *in vitro* (83). Moreover, these effector cells produced significant levels of INF-γ upon co-cultivation with the target cells (83). *In vivo* assessments of this study entailed preclinical mouse models of ovarian cancer established using subcutaneously administered SKOV3 cells (83). According to the results, the administration of EpCAM-redirected CAR-Ts into mouse models reduced the size of the established tumors and remarkably slowed down tumor outgrowth in comparison with the control group (83). Overall, these researchers suggested that EpCAM might act as a potent target antigen for CAR-T therapy of ovarian cancer; even though more in-depth assessments are required (83). In addition to this study, Herbel and colleagues reported the results of an ongoing study investigating the expression of THY1 and EpCAM on the surface of ovarian cancer cells and the suitability of these target antigens for ovarian cancer CAR-T therapy with the aim of addressing the “on-target off-tumor” toxicity associated with this platform of cancer immunotherapy (84). In detail, THY1 is a cell surface protein present in fibroblasts and hematopoietic stem cells (84). First, Herbel et al. assessed the expression of THY1 and EpCAM on a number of primary ovarian cancer patient-derived samples using high-content imaging (84). The results of this investigation indicated that THY1-EpCAM-redirected CAR-Ts mediated tumoricidal functionality against target cells *in vitro* (84). Overall, these researchers added that they are planning to assess the efficacy and safety of these multi-targeting THY1-EpCAM-redirected CAR-Ts in animal models of ovarian cancer. Until then, more substantiated data in regard to the efficacy of these CAR-Ts remains to be obtained (84). In 2020, Qin et al. reported that murine EpCAM-redirected CAR-Ts can mediate lung attack and lethality in immunocompetent preclinical mouse models of breast cancer established using the breast cancer cell line 4T1 (85). In detail, these CAR-Ts mediated effective antitumor activity and produced INF-γ when co-cultured with different EpCAM-expressing cancer cell lines, including 4T1 and MC38; however, they did not demonstrate cytotoxicity against the EpCAM-deficient normal fibroblast control cells NIH-3T3, indicating the antigen-specific tumoricidal activity of these effector cells (85). These results were consistent with those of the previously published studies investigating EpCAM-redirected CAR-Ts in several types of solid tumors including peritoneal carcinomas, prostate cancer, and colorectal cancer (86–88). In reference to the *in vivo* assays, EpCAM-redirected CAR-Ts significantly reduced tumor burden in preclinical mouse models in comparison with control T cells (85). It is worth mentioning that these researchers also evaluated the antitumor response of these CAR-Ts in mouse models of colon cancer established using the MC38 colon cancer cell line, and demonstrated that these CAR-Ts reduced tumor outgrowth and extended the survival of the tumor models (85). However, these researchers indicated that their murine EpCAM-redirected CAR-Ts recognized and became activated by EpCAM expressed on non-malignant tissues leading to on-target off-tumor toxicities (85). Moreover, Qin et al. demonstrated an expression pattern of EpCAM in the lung bronchioles (85). It was also demonstrated that alveolar EpCAM expression in normal lung tissues results in the recruitment of EpCAM-redirected CAR-Ts and their activation in the mentioned sites leading to CAR-T-mediated lung inflammation and tissue damage (85). Such data highlight the importance of broader research, both in the preclinical and clinical stages, in this field to prevent the occurrence of such unwanted adverse events (85).

### 4.11 Folate receptor β (FRβ)

Lynn and colleagues reported the applicability of FRβ-redirected CAR-Ts for the elimination of tumor-associated macrophages in ovarian cancer (89). In the TME, TAMs have been recognized as elements supportive of tumor progression (30, 89). In the context of ovarian cancer, a dismal prognosis has been attributed to the presence of TAMs (89). FRβ is known as a surface-expressed molecule that plays an important role in folic acid uptake (89). In a variety of individuals with cancer and preclinical cancer animal models, the expression of FRβ has been documented (89). Moreover, Lynn and colleagues have also reported the expression of this GPI-linked molecule in M2-polarized macrophages (89). In this regard, these researchers developed FRβ-redirected CAR-Ts using a high-affinity targeting domain specific for FRβ (89). It was demonstrated that FRβ-redirected CAR-Ts were capable of mediating cytolytic effects and secreting INF-γ against M2 polarized macrophages, and upon co-cultivation of FRβ-redirected CAR-Ts with the SKOV3 cell line (deficient in the expression of FRβ), the engineered T cells mediated tumoricidal effects while M2 polarized macrophages were present (proficient in the expression of FRβ) (89). Based on the findings by Lynn and colleagues that FRβ exhibits an elevated expression level in TAMs isolated from individuals with ovarian cancer, these researchers suggested that FRβ-redirected CAR-Ts could be of therapeutic benefit for the elimination of such TAMs (89). To further validate these findings, these researchers established ID8-based ovarian cancer mouse models and set out to assess the tumoricidal efficacy of FRβ-redirected CAR-Ts (referred to as CL10) *in vivo* (89). Of note, ID8 is a murine cell line of ovarian epithelial that is biologically similar to human ovarian cancer (89). Upon administration of CL10 into the animal models, the researchers reported CAR-T-mediated elimination of FRβ-positive TAMs and suppression in the outgrowth of the established tumors (89). Moreover, the researchers proposed that future investigations could focus on the synergistic effects of CL10 coupled with CAR-Ts redirected against a TAA or TSA of interest since it has been experimentally evident that CAR-T-mediated elimination of TAMs could lead to better tumor control in solid tumors (89). Lynn and colleagues also published a paper on FRβ-redirected CAR-T-mediated targeting of AML blasts, based on the findings that FRβ expression has been evident in a high percentage of AML samples (90). Briefly, these researchers genetically modified the FRβ-negative cell line C3023 (of ovarian cancer origin) to stably express FRβ (90). Upon co-cultivation of the FRβ-redirected CAR-Ts (equipped with an scFv called *m909* as the targeting domain) with the engineered C3023 cell line alongside other FRβ-positive AML cell lines, it was demonstrated that the CAR-Ts were capable of mediating antigen-dependent tumoricidal effects against the target cells (90). Moreover, the investigators also established xenograft mouse models of FRβ-positive human AML based on the THP1 cell line (90). Upon administration of FRβ-redirected CAR-Ts into the preclinical subjects, suppression of tumor progression was evident (90). One of the main concerns of selecting FRβ as a target antigen is its possible shared expression in CD34-positive hematopoietic stem and progenitor cells (HSCs). Lynn and colleagues investigated this matter and elucidated that FRβ-redirected CAR-Ts did not mediate cytolytic reactions against HSCs (90). These researchers also evaluated a strategy to improve the recognition of FRβ-positive cells by FRβ-redirected CAR-Ts and demonstrated that all-*trans* retinoic acid could elevate FRβ expression levels in AML cell lines (THP1 and MV411) (90). According to another study, Lynn et al. elucidated how the affinity of the CAR target antigen recognition domain could be a factor of paramount importance in the context of CAR-T therapy, and how transient CAR expression policies could minimize the health risks associated with stable CAR expression in T cells (91). Briefly, these researchers reported the isolation of a high-affinity scFv (2.48 nM) specific for FRβ and demonstrated that CAR-Ts equipped with this scFv showed pronounced tumoricidal capacity against FRβ-positive AML cells in comparison with FRβ-redirected CAR-Ts that harbored a low-affinity scFv as their targeting domain, in preclinical experiments (91). Since the FRβ-redirected CAR-Ts exhibited cytolytic activities against mature CD14-positive monocytes and to minimize the risk of CAR-T-mediated cytolysis of mature myeloid lineage, Lynn et al. proposed the strategy of developing mRNA-based FRβ-redirected CAR-Ts and demonstrated that these engineered T cells managed to maintain their tumoricidal efficacy in preclinical experimental conditions (91). Studies such as those discussed herein highlight the importance of CAR-T-mediated targeting of FRβ in solid tumors, such as ovarian cancer, as well as myeloid-derived malignancies; however, clinical validation of these findings could shed more light on the suitability of FRβ as a cancer immunotherapy target antigen (91).

### 4.12 Folate receptor α (FRα)

FRα (alternatively known as FOLR1) could be considered a favorable target antigen in the CAR-T therapy of ovarian cancer owing to its elevated level of expression in a high percentage of ovarian cancer cases (92, 93). Alongside this oncological indication, FRα overexpression has been documented in a variety of solid tumors, inclusive of lung cancer, mesothelioma, and breast cancer, as well as other carcinomas (92, 94, 95). Moreover, its localized expression in epithelial cells renders it unreachable to FRα-specific therapeutics, and its negligible expression in normal tissues further validates its possible suitability as a CAR-T therapy target antigen (96). One of the earliest studies on this topic was conducted by Kershaw and colleagues as they genetically manipulated autologous T cells for the expression of chimeric receptors redirected against FRα using an scFv fused to the intracellular domain of the Fc gamma receptor (97). Individuals with ovarian cancer were divided into two cohorts as eight of them underwent T cell therapy with high-dose IL-2 (cohort I) and six underwent pre-treatment with dual-specific T cells scheduled to be followed by allogeneic peripheral blood mononuclear cells (PBMCs) for immunization (cohort II) (97). The findings indicated no tumor shrinkage, as five individuals in the first cohort showed attention-requiring toxicities (grade 3/4; attributable to high-dose IL-2) whereas those in the second cohort exhibited milder signs of toxicities (grade 1/2) (97). Further examination of the administered T cells demonstrated poor tumor-site trafficking in most of the patients in cohort I (97). Moreover, Kershaw and colleagues reported a sharp decline in the number of administered T cells in most patients, which was attributed to the emergence of an inhibitory element in some patients (97). Our speculation for this occurrence is the formation of neutralizing antibodies against the scFv incorporated into the CAR construct of these engineered T cells (97). This scFv was derived from the murine mAb *MOv18* (97, 98). Examination of the treated patients’ sera for the presence of such neutralizing antibodies would have possibly elucidated the reason for such poor *in vivo* CAR-T persistence.

It has been evident that the FRα-specific humanized mAb *farletuzumab* has strong tumor growth suppression in animal-based models of human solid tumors, and Lin and colleagues reported that such tumoricidal effects are exerted through antibody-dependent cellular cytotoxicity (ADCC) by conducting an investigation using mouse models of ovarian cancer (99). Moreover, *farletuzumab* has also been assessed in various clinical settings for therapeutic purposes in individuals with ovarian cancer (NCT03386942, NCT02289950, etc.) (99). An scFv derived from such mAbs could be of interest for the development of FRα-redirected CAR-Ts to overcome the issues of murine scFv immunogenicity. In contrast with the MOv18-derived scFv, Song and colleagues incorporated a fully human FRα-specific scFv (named C4) into a CAR construct to develop FRα-redirected CAR-Ts (100). *In vitro* assays indicated that C4-based CAR-Ts were able to mediate tumoricidal effects against FRα-positive ovarian cancer cell lines, SKOV3 and OVCAR5, upon co-cultivation, alongside secreting elevated levels of INF-γ (100). Moreover, Song and colleagues established ovarian cancer mouse models by intraperitoneal injection of SKOV3 cells into NGC mice and reported that intravenous administration of C4-based CAR-Ts resulted in significant tumor volume reduction (100). Furthermore, a comparison of C4-based CAR-Ts with MOv19-based CAR-Ts (whose targeting domain is based on a MOv19-derived scFv; a mAb similar to MOv18) showed that these two products mediated comparable cytolytic reactions against SKOV3 and A1847 cell lines alongside secreting comparable levels of INF-γ (100). Moreover, these two CAR-T products also mediated comparable therapeutic effects in mouse models with established tumors (100). Upon the co-cultivation of each of these CAR-T products with HEK293T and IOSE6 (with minimal levels of FRα expression), it was demonstrated that C4-based CAR-Ts secreted lower levels of INF-γ and TNF-α in comparison to those secreted by MOv19-based CAR-Ts (100). This lower antigen reactivity of C4-based CAR-Ts could be attributed to the lower affinity of their scFv to FRα, which could be a factor of paramount importance in reducing on-target off-tumor effects of CAR-Ts towards healthy tissues with physiological levels of target antigen expression (100). According to another study, Xu and colleagues developed FRα-redirected CAR-Ts and evaluated their characteristics in the presence of a panel of cytokines (IL-2, -7, -15, -18, and -21) in preclinical conditions (101). The first three cytokines supported the expansion of CAR-Ts *ex vivo* as compared to IL-18, IL-21, or the absence of any cytokine treatment (101). Moreover, the highest degree of CAR-T differentiation was observed in the IL-2 treatment group whereas CAR-Ts in the IL-7 and IL-21 treatment groups more shifted towards stem cell-like memory T cells and less differentiated populations, respectively (101). In terms of tumoricidal effects and cytokines secretion, CAR-Ts in the IL-2 and IL-15 treatment groups were superior to others *in vitro*; however, CAR-Ts in the former cytokine treatment group exhibited the weakest level of tumoricidal effects *in vivo*, as an incremental pattern was observed in the antitumor efficacy of CAR-Ts in IL-15 and IL-21 cytokine groups (101). Ultimately, Xu and colleagues concluded that IL-7 and IL-15 are the more suitable options for supporting the expansion of CAR-Ts *ex vivo* whereas IL-15 and IL-21 are the preferred cytokines for *in vivo* administration following adoptive transfer of CAR-Ts (101).

One of the strategies proposed by researchers for overcoming the limitations of CAR-T-mediated on-target off-tumor toxicities is the development of CAR-Ts equipped with AND or OR gates (42, 102). However, the practicality of this approach is somewhat questioned by the set of target antigens such strategies are developed upon (42, 102). In the context of HGSC, Banville and colleagues conducted an investigation to identify antigens that could be targeted via combinatorial CAR-T platforms by investigating the simultaneous expression of FRα, mesothelin, and CA125 in different HGSC tumor samples (103). Briefly, all of the mentioned target antigens exhibited more than 90% expression in the tumor samples whereas the first two were also substantially expressed by normal tissues at the transcript level (103). CA125, mesothelin, and FRα had the highest to lowest expression rates, respectively, in tumor samples and percentages of malignant cells at the protein level (103). These researchers reported that an OR gate CAR-T platform based on CA125 and mesothelin would be the most applicable choice as a high percentage of malignant cells in around 60% of the assessed tumor samples were proficient in the expression of either antigen (103). Conclusively, Banville and colleagues asserted that an OR gate CAR-T platform based on mesothelin and CA125 would be capable of targeting a high proportion of malignant cells in most clinical cases (103). However, a personalized AND or OR gate must be developed for each patient undergoing CAR-T therapy since the expression pattern of each of these antigens could differ in patients with different stages of advanced ovarian cancer.

### 4.13 MUC16

MUC16 (alternatively known as mucin 16) is a heavily glycosylated protein with important roles in cellular maintenance and epithelial protection; however, its tumor-associated expression has been correlated with tumor cell progression and migration in numerous oncological indications (104). The expression of MUC16 has been documented in a high percentage of ovarian cancer cases (105). The full-length protein of MUC16 is cleaved as a fragment is released into the bloodstream (known as CA125; which has been leveraged for diagnostic purposes) and the remainder is left displayed over the cell surface (known as ectoMUC16; which has been exploited for therapeutic purposes by researchers) (104, 105). CAR-T-mediated targeting of MUC16 has been investigated in multiple studies which are briefly discussed in this section. For instance, Chekmasova and colleagues derived an scFv from the mAb *4H11* by fusing V_H_ and V_L_ by means of a flexible linker peptide (106). This scFv was applied as the targeting domain of 2^nd^-generation CAR-Ts (whose CAR construct was based on the CD28 co-stimulatory and CD3ζ activation domains) redirected against ectoMUC16 (106). Upon co-cultivation of the generated CAR-Ts with ectoMUC16-proficient cell lines, SKOV3 and OVCAR3, it was demonstrated that the CAR-Ts were capable of mediating specific tumoricidal reactions in a dose-dependent fashion, alongside exhibiting antigen-dependent proliferation (106). Moreover, these CAR-Ts secreted significantly elevated levels of IL-2 and INF-γ over the course of a two-day co-cultivation (106). To further validate these findings, Chekmasova and colleagues developed allogeneic (from a healthy donor) and autologous ectoMUC16-redirected CAR-Ts and demonstrated that these engineered T cells managed to enforce cytolytic reactions against primary patient-derived tumor cells proficient in the expression of ectoMUC16 (106). These researchers also established ovarian cancer SCID-Beige mouse models (based on the OVCAR3 cell line) and demonstrated that intravenous or intraperitoneal administration of the ectoMUC16-redirected CAR-Ts resulted in tumor regression and prolonged survival of the animal models, without any significant difference between CAR-T delivery routes (106). Ultimately, Chekmasova and colleagues asserted that such encouraging outcomes could be the foundation of future clinical investigations with patients diagnosed with MUC16-positive ovarian cancer (106). Chekmasova and co-researchers conducted another study to investigate the antitumor efficacy of ectoMUC16-redirected CAR-Ts engineered to secrete IL-12 in preclinical mouse models of ovarian cancer (107). Briefly, T lymphocytes were engineered to express ectoMUC16-redirected CARs (based on the 4H11 scFv) and to secrete IL-12 (107). The researchers established ectoMUC16-positive ID8-based mouse models and demonstrated that adoptive transfer of IL-12-secreting ectoMUC16-redirected CAR-Ts resulted in complete elimination of the peritoneal established tumor lesions, in comparison with conventional ectoMUC16-redirected CAR-Ts (107). Moreover, it was elucidated that there were higher numbers of IL-12-secreting ectoMUC16-redirected CAR-Ts in the peritoneum of the treatment subjects which also correlated with elevated rates of endogenous T lymphocyte recruitment to the tumor lesions, in comparison with the other treated groups (107). The investigators also asserted that such antitumor effects were not dependent on pre-treatment lymphodepletion and that treatment was regarded as well-tolerated (107). Ultimately, the researchers concluded that ectoMUC16-redirected CAR-Ts could successfully eliminate transplanted ovarian tumors in mouse models, as such favorable therapeutic effects could also be further improved while the CAR-Ts were engineered to secrete IL-12 (107). Koneru and colleagues conducted a similar study in which 4H11-based ectoMUC16-redirected CAR-Ts were developed, which were further engineered to secrete IL-12 (108). Aside from favorable *in vitro* outcomes, IL-12-secreting ectoMUC16-redirected CAR-Ts mediated strong tumoricidal responses in xenograft mouse models of ovarian cancer (based on SCID-Beige mice) which led to extended survival of the treatment subjects and persistence of the effector cells alongside elevated levels of INF-γ (108). Based on these favorable findings, a Phase I clinical investigation was initiated to evaluate the safety and therapeutic efficacy of these IL-12-secreting CAR-Ts (which were also equipped with a safety switch for their at-will elimination following administration) in individuals with ectoMUC16-proficient ovarian carcinoma for the first time (108, 109). Yeku and colleagues suggested that a potential counterstrategy against antigen-dependent tumor relapse (antigen loss or downregulation) would be to engage other parties of the immune system to augment the antitumor responses, aside from those mediated by CAR-Ts (110). These researchers investigated whether genetic manipulation of ectoMUC16-redirected CAR-Ts for the secretion of IL-12 could have a therapeutic advantage against peritoneal ovarian tumors with high or low levels of ectoMUC16 expression *in vitro* and in preclinical animal models (110). The researchers reported that these CAR-Ts were able to mediate tumoricidal responses against ID8 cells with high or low levels of ectoMUC16 expression, with IL-12-secreting ectoMUC16-redirected CAR-Ts exhibiting more effective tumoricidal reactivity in comparison with that of conventional ectoMUC16-redirected CAR-Ts (110). Moreover, peritoneal tumor mouse models were established by intraperitoneal injection of tumor cells into C57BL/6 mice, and it was demonstrated that 12-secreting ectoMUC16-redirected CAR-Ts prolonged the survival of the treated mice (for whose tumor transplantation, a ratio of 1:1 of high ectoMUC16-expressing tumor cells: low ectoMUC16-expressing tumor cells were used) upon adoption transfer (110). Furthermore, it was elucidated that treatment with 12-secreting ectoMUC16-redirected CAR-Ts culminated in the amplification of mature dendritic cells of the treated subjects’ peritoneum, as an incremental pattern was also observed in the TCR clonality of this experimental group (110). Ultimately, Yeku and colleagues asserted that the application of 12-secreting CAR-Ts could be a potential strategy to counteract the heterogeneity of solid tumors (110).

According to another investigation, Yeku and colleagues attempted to evaluate strategies aimed at overcoming the immunosuppressive nature of the ovarian cancer TME (111). Briefly, these researchers further modified ectoMUC16-redirected CAR-Ts to secrete IL-18 and reported that these CAR-Ts were able to significantly prolong the survival of ovarian cancer syngeneic mouse models, with high and low tumor burdens, upon adoptive transfer (111). Another counterstrategy evaluated by Yeku et al. was to engineer CAR-Ts for the secretion of PD-1-specific scFvs which culminated in augmented antitumor efficacy in mouse models of ovarian cancer alongside prolonging the persistence of the CAR-Ts in a way that they resisted tumor rechallenge (111). Moreover, it was demonstrated that this approach resulted in an increase in the engagement of the endogenous immune elements (111). Yeku et al. also developed ectoMUC16-specific TRBAs and reported that they mediated significant tumor shrinkage in xenograft mouse models of ovarian cancer (111). Clinical assessment of such CAR-Ts and TRBAs could further validate their therapeutic efficacy in patients with ectoMUC16-positive ovarian cancer (111). According to another preclinical investigation, Li and colleagues generated a lentivirally transduced dual CAR-T product redirected against PD-L1 and ectoMUC16 (PD-L1-ectoMUC16-redirected CAR-Ts) and demonstrated that these engineered T lymphocytes were able to enforce pronounced tumoricidal effects against OVCAR3 cells alongside secreting elevated levels of IL-2, IFN-γ, and TNF-α upon co-cultivation with the mentioned cell line (112). Moreover, these researchers also developed OVCAR3-based mouse models of ovarian cancer and reported that adoptive transfer of these dual CAR-Ts resulted in the extended survival of the animal models, which were two- to four-fold more pronounced than those induced by CAR-Ts redirected against either PD-L1 or ectoMUC16 (112). Such findings necessitate the evaluation of the safety profile and therapeutic efficacy of PD-L1-ectoMUC16-redirected CAR-Ts in clinical settings. Recently, O’Cearbhaill and colleagues initiated a Phase I clinical investigation (NCT02498912) to assess the safety and therapeutic efficacy of IL-12-secreting ectoMUC16-redirected CAR-Ts (equipped with a truncated EGFR-based safety switch) administered through the intravenous or intraperitoneal route into eighteen individuals with ectoMUC16-proficient serous carcinoma of the ovary with prior lines of therapy (113). Briefly, patients were put into five cohorts (I-V); as patients in cohorts I to IV underwent CAR-T treatment with four different doses (ranging from 3 × 10^5^ to 1 × 10^7^ CAR-Ts/kg), and those in cohort V were lymphodepleted with Cy/Flu before CAR-T treatment (with the 3 × 10^6^ CAR-Ts/kg dose) (113). According to the findings, no significant clinical signs of on-target off-tumor toxicities nor any DLTs were documented in the evaluated patients of the first four cohorts, as two out of three patients treated in cohort V experienced DLTs (113). Moreover, CRS was reported to occur at all investigated doses (113). Ultimately, According to the *Response Evaluation Criteria in Solid Tumors (RECIST)* criteria, the best documented response to the CAR-T treatment was stable disease (113). Conclusively, the researchers asserted that they aim to increase the *in vivo* persistence of these CAR-Ts by combining them with PD-1-specific therapy (113). Such findings highlight the importance and possible suitability of MUC16 as a CAR-T therapy target antigen; however, hurdles must be recognized and arduously overcome to better the therapeutic responses achieved in ovarian cancer patients.

### 4.14 NKG2D ligands (NKG2DLs)

NKG2D is surface-expressed by a variety of immune cells, including cytotoxic T lymphocytes, γδ T lymphocytes, and Natural Killer (NK) cells, in humans (114). In T lymphocytes, NKG2D engages with DAP10 only to trigger co-stimulatory signals for TCRs, whereas, in NK cells, it provides main activation signals (115–117). In various types of solid tumors, such as ovarian cancer, NKG2DLs (such as MICA/B, ULBP, Letal, etc.) are frequently expressed; fortunately, mRNAs for their ligands are reported to exist in healthy tissues, but their surface expression is restricted or negligible (118–121). Therefore, researchers have suggested that NKG2DLs could be leveraged for therapeutic purposes without causing serious risks of toxicities towards healthy tissues (122). According to a study, Barber and colleagues devised a chimeric receptor by fusing NKG2D to the CD3ζ of the TCR, and genetically engineered T cells to express this receptor, and demonstrated that the engineered T cells exhibited tumoricidal reactivity and secreted proinflammatory cytokines upon engagement of their engineered receptors with NKG2DLs (which were surface-expressed by more than 80% of human ovarian cancer biological samples, and the ovarian cancer cell lines A2780 and A2008) (122). Barber et al. attempted to further validate their engineered T cells as these researchers established GFP-positive ID8 cell line-based mouse models of ovarian cancer, and treated the animal subjects with their engineered T cells (5 × 10^6^ cells) a week following tumor establishment (122). 2 months later, the animal models were sacrificed to measure tumor burden and assess the antitumor efficacy of the engineered T cells (122). According to the results, the engineered T cells were capable of mediating ID8-based tumor outgrowth suppression, as measured by a decline in the percentage of GFP-positive cells and the number of tumor lesions in the animal peritoneum (122). Ultimately, Barber and colleagues suggested that their findings indicate that T cells endowed with NKG2D-based chimeric receptors are potential therapeutic options for ovarian cancer; however, broader investigation at the clinical level is warranted for such assertions (122). Barber and colleagues conducted another investigation to further elaborate on the applicability of T cells engineered with NKG2D-based chimeric receptors (designed in the previous study) by determining if adoptive transfer of these T cells could prolong the survival of ovarian cancer animal models, and whether the immune system of the treatment subjects could mount responses to antigens associated with ovarian cancer (123). Briefly, B6 mouse models of the GFP-positive ID8 cell line were established, and then treated with 5 × 10^6^ engineered T cells (or control T cells) via intraperitoneal administration (123). According to the results, treatment with the engineered T cells significantly prolonged the survival of the animal models in comparison with the control group (123). Moreover, the investigators further elucidated that the tumor-free surviving treatment subjects managed to develop protective immune reactions against ovarian cancer (which include memory CD8-positive T cell and CD4-positive T cell reactions) since they rejected another ovarian tumor development with the ovarian cancer cells, 225 days following the initial time of tumor establishment (123). Furthermore, the investigators asserted that the complete tumoricidal capacity of the engineered T cells was also dependent on the secretion of perforin and IFN-γ, as well as GM-CSF (123).

According to another study, Song and colleagues generated NKG2DL-redirected CAR-Ts by incorporating the extracellular domain of NKG2D into a CAR construct (based on 4-1BB and CD3ζ costimulatory and activation domains, respectively), and set out to evaluate the feasibility of augmenting the sensitivity of NKG2DL-positive tumor cells by pharmacologically increasing the expression rate of NKG2DL (124). However, these researchers reported that the expansion of the NKG2DL-redirected CAR-Ts was hindered, in comparison with that of the control T cell groups, mainly due to the expression of NKG2DLs by the NKG2DL-redirected CAR-Ts, which would consequently result in their fratricide (124, 125). To overcome this issue, the investigators increased the duration of *in vitro* expansion (124). According to the results of the *in vitro* experiments, upon the co-cultivation of the NKG2DL-redirected CAR-Ts with NKG2DL-positive ovarian cancer cell lines, A1847 and OVCAR5, the effector cells mediated strong cytolytic reactions against the target cells (at the effector: target ratio of 3, 1, and 0.3), while they managed to spare the AE17 mesothelioma cell line deficient in the expression of NKG2DLs (124). Next, Song et al. treated the ovarian cancer cell lines A2780, PEO-1, and OVCAR5 (which expressed low to moderate levels of NKG2DLs) with 2 mM of sodium valproate (which is a histone deacetylase inhibitor), and demonstrated that this treatment increases the surface expression of NKG2DLs on these cells, which consequently culminates in their increased susceptibility to NKG2DL-redirected CAR-T treatment (as was evident from the elevated levels of secreted IFN-γ in their co-culture) (124). Ultimately, Song et al. concluded that the application of sodium valproate with NKG2DL-redirected CAR-T treatment could have therapeutic benefits for patients with ovarian cancer; however clinical assessment would have to validate the safety and clinical applicability of this strategy. According to another investigation, Spear and colleagues developed T cells engineered to express NKG2D-based CAR constructs (referred to as chNKG2D-CAR-Ts) and demonstrated how adoptive transfer of these cells resulted in sufficient antitumor effects against NKG2DL-positive and NKG2DL-negative ovarian cancer tumors and how chNKG2D-CAR-T treatment conferred antitumor immunity against NKG2DL-deficient ovarian cancer cells (126). Briefly, these researchers established ID8 cell line-based ovarian cancer mouse models and reported remarkable chNKG2D-CAR-T-mediated tumoricidal effects against populations of ovarian tumors that expressed fluctuating levels of NKG2DL (from 7 to 50%) (126). However, the researchers asserted that these antitumor responses were strongly dependent on the expression rate of NKG2DL and the percentage of NKG2DL-positive tumor cells within a tumor bulk (126). Moreover, Spear and colleagues evaluated whether successful treatment with chNKG2D-CAR-T could provide host immunity to animal models against an ID8-based ovarian cancer rechallenge (126). To this aim, first, GFP-positive ID8 cells were modified with a Rae1 shRNA (ID8/GFP-shRae1) to render them NKG2DL-deficient (of note, this cell line is deficient in the expression of Mult1 or H-60 NKG2DLs) (126). Next, chNKG2D-CAR-Ts were intraperitoneally administered into the same animal models free of the ID8-based ovarian tumors, and, eight weeks following treatment, it was demonstrated that the formation of ovarian tumor lesions as well as tumor progression were suppressed in these mouse models, compared with the control group (126). According to another study, Spear and colleagues demonstrated that adoptive T cell therapy results in conferring host T cell-based immunity that cooperates in the eradication of tumor lesions, and the formation of tumor-reactive immune reactions (127). Briefly, upon the administration of NKG2DL-redirected CAR-Ts into ID8-based preclinical mouse models, the researchers reported a CAR-T-induced increase in the population of tumor-resident endogenous CD4-positive T cells and CD8-positive T cells in a fashion dependent on CXCR3, as well as expansion in the population of tumor- and lymph-resident tumor-reactive endogenous CD4-positive T cells (127). Moreover, the researchers reported a CAR-T-induced increase in antigen presentation to CD4-positive T cells which was dependent on CAR-T-mediated secretion of INF-γ and GM-CSF (127). Ultimately, the researchers asserted that efficient tumor eradication mediated by NKG2DL-redirected CAR-Ts relied on the presence of endogenous CD8-positive T cells (127).

Toxicities associated with the activation and expansion of CAR-Ts following administration are a result of cytokine release (128). In the context of NKG2DL-redirected CAR-Ts, Ng and colleagues attempted to overcome this clinical hindrance by designing a 2^nd^ generation CAR construct based on the extracellular domain of NKG2D, as the targeting domain, and the 4-1BB co-stimulatory domain and the DAP12 activation domain (128). In a comparative view, DAP12-based NKG2DL-redirected CAR-Ts secreted lower levels of INF-γ, TNF-α, and IL-2, and their proliferation capacity was lower in response to repeated antigen encounter while mediating tumoricidal effects *in vitro*, without any observable difference between DAP12-based NKG2DL-redirected CAR-Ts and CD3ζ-based NKG2DL-redirected CAR-Ts in the context of mediating cytolytic reactions (128). Ng and colleagues further established NSG mouse models based on the HCT116 colorectal cancer cell line and reported that the administration of DAP12-based NKG2DL-redirected CAR-Ts and CD3ζ-based NKG2DL-redirected CAR-Ts mediated similar tumoricidal effects that led to the elimination of the established tumors (128). Of note, high mortality rates were reported only in the xenograft group treated with the CD3ζ-based NKG2DL-redirected CAR-Ts which was a result of graft-versus-host disease (GvHD), as higher levels of serum cytokines were documented in this experimental group (128). Ultimately, the investigators concluded that designing CARs based on the DAP12 activation domain might be a feasible strategy for minimizing the adverse events associated with CAR-T-mediated CRS (128).

Despite the fact that various studies reported the expression of NKG2DL or PD-L1 in numerous types of oncological indications, whether the simultaneous expression of these two antigens is present in different tumors is less explored (129). In this regard, Jiang and colleagues investigated the expression of NKG2DL and PD-L1 in human ovarian cancer tissue samples and demonstrated that almost 80% of the samples exhibited the co-expression of the mentioned antigens (129). Briefly, these researchers designed a unique dual CAR circuit that was based on two separate CAR constructs; one based on the extracellular domain of NKG2D fused to the DAP12 activation domain (which provides the principal activation signals), and the other based on a high-affinity PD-L1-specific scFv fused to the co-stimulatory domain of 4-1BB (which provides auxiliary signals necessary for efficient activation of CAR-Ts following antigen encounter) (129). The appliance of this high-affinity scFv was taken into consideration as a potential strategy to enable dual CAR-Ts to recognize tumor cells with low-level expression of PD-L1 (129). According to the results of the *in vivo* experiments, adoptive transfer of the dual CAR-Ts resulted in the elimination of ovarian cancer-established tumors with metastatic peritoneal lesions in preclinical animal models (129). A dual CAR-T platform, such as the one developed by Jiang and colleagues, might offer therapeutic benefits for the treatment of metastatic peritoneal tumor lesions proficient in the expression of NKG2DL and PD-L1 (129). According to another study, Wang and colleagues investigated whether treatment of the ovarian cancer cell line SKOV3 with *romidepsin* (an anticancer agent utilized in the treatment of T-cell lymphomas) could increase their susceptibility to NKG2DL-redirected CAR-T treatment through increasing NKG2DL expression (130). The results of the *in vitro* experiments implicated increased surface expression of NKG2DL in the SKOV3 cells which consequently resulted in the increased tumoricidal effects of the NKG2DL-redirected CAR-Ts against these cells (accompanied by elevated levels of secreted INF-γ) (130). Ultimately, Wang and colleagues concluded that increasing the expression rate of the CAR-T-targeted antigens could enhance the antitumor efficacy of this platform of immunotherapy; however in-depth clinical evaluations of such strategies are warranted for further elucidation (130).

### 4.15 Mesothelin

Mature mesothelin is a 40 kDa membrane-expressed protein which is the result of a 71 kDa Furin-cleaved protein known as the precursor mesothelin (131). Primarily introduced in the 1990s, this protein exhibits negligible expression levels in the cells of the peritoneum and pleura, whereas its elevated expression in malignant mesothelioma patients has been confirmed in a high percentage of tumor samples (131, 132). Despite its undeciphered, and probably inessential, physiological function in normal cells, its elevated tumor-associated expression has been correlated with tumor aggressiveness and progression (133–135). Ever since its discovery, mesothelin has been an interesting target antigen in investigations relating to cancer immunotherapy. In the context of ovarian cancer CAR-T therapy, mesothelin can be named as the most researched target antigen, which has been the subject of various CAR-T-based preclinical and clinical investigations, which are briefly discussed in this section.

In 2012, Lanitis and colleagues conducted an investigation to address the issue of poor CAR-T persistence *in vivo* induced by the formation of neutralizing antibodies against the targeting domain of CAR-Ts derived from animal-based mAbs (136). To this aim, these researchers proposed that using fully human targeting domains can be taken into consideration in the construction of CAR molecules; therefore, they applied a fully human scFv, called P4, specific for human mesothelin as the antigen-recognition domain of their CAR construct (136). According to the results of the *in vitro* experiments, these mesothelin-redirected CAR-Ts secreted proinflammatory cytokines and mediated strong tumoricidal reactions upon their co-cultivation with mesothelin-positive cells, as this functionality was not suppressed by the presence of recombinant mesothelin or its cancer cell-secreted form (136). Moreover, Lanitis and colleagues developed human ovarian cancer xenograft mouse models by subcutaneous inoculation of the A1847 cell line into NSG mice and reported that intratumoral administration of mesothelin-redirected CAR-Ts culminated in tumor outgrowth suppression while the animals were under soluble mesothelin treatment (136). Ultimately, Lanitis et al. concluded that mesothelin-redirected CAR-Ts, whose targeting domains are based on the mentioned fully human scFv, can potentially eliminate mesothelin-positive tumors in preclinical conditions while being capable of overcoming the issue of anaphylaxis induced by the immunogenic targeting domains of CAR constructs (136). In 2016, Tanyi and colleagues reported the results of a Phase I clinical investigation (NCT02159716) assessing the tumoricidal efficacy of 2^nd^-generation mesothelin-redirected CAR-Ts intravenously administered to six individuals with ovarian cancer (137). Briefly, these autologous CAR-Ts benefited from a mesothelin-specific scFv derived from the SS1 mAb (of murine origin), the 4-1BB co-stimulatory domain, and the CD3ζ main activation domain (137). Moreover, four individuals underwent a single round of CAR-T therapy with the 3 × 10^7^/m^2^ dosage whereas two other individuals received a single round with a higher dose (3 × 10^8^/m^2^) (137). Of note, this treatment scheme entailed CAR-T treatment with or without the use of lymphodepleting chemotherapy (137). According to the results, no CRS nor serious adverse events related to the administration of the CAR-Ts were observed, as only two patients experienced grade 3 adverse events (such as abdominal discomfort) and one experienced grade 3 to 4 adverse events (namely, pleural effusion and rapid and shallow breathing, as well as shortness of breath) (137). Moreover, the investigators reported higher *in vivo* expansion rates for the CAR-Ts of the patients treated with the higher dosage and lymphodepletion (137). Examination of tumor samples of three out of four patients (75%) revealed the presence of the mesothelin-redirected CAR-Ts, which confirmed their sufficient infiltration, with their tumoricidal efficacy being evident by the elimination of pleural tumor cells three weeks following treatment without lymphodepleting regimens (137). Ultimately, based on the RECIST criteria, the investigators reported that all six patients achieved stable disease (137). The authors asserted that such findings highlight the safety and applicability of mesothelin-redirected CAR-Ts in individuals with serous carcinoma of the ovary and further clinical evaluations might benefit from these outcomes (137).

According to another investigation, Tanyi and colleagues, for the first time, reported the development of CRS in the pleural cavities of a middle-aged female individual with serous ovarian carcinoma who had undergone mesothelin-redirected CAR-T therapy (3 × 10^7^/m^2^) without any lymphodepleting regimen (138). This occurrence was characterized by elevated levels of IL-6 and a high population of mesothelin-redirected CAR-Ts in the patient’s pleural fluid; three weeks following CAR-T administration, this severe toxicity was resolved with the application of the IL-6-specific mAb *tocilizumab* (138). These researchers suggested that the formation of an environment by the patient’s pleural fluid in which CAR-Ts and cancer cells could interact could be the underlying mechanism for this event (138). According to another study, Gruzdyn and colleagues lentivirally transduced primary T cells to develop scFv-based mesothelin-redirected CAR-Ts, and they reported that the CAR-Ts significantly secreted elevated levels of granzyme B and INF-γ upon their co-cultivation with the mesothelin-positive SKOV3 ovarian cancer cell line (139). Moreover, these researchers demonstrated that increasing the effector:target cell ratio from 10:1 to 20:1 resulted in higher percentages of CAR-T-mediated SKOV3 cell lysis (from ~40 to 61%) (139). Experimental evidence has suggested that ovarian tumor cells evade the immune system by means of secreting inhibitory cytokines, including IL-10 (140). In line with the previously discussed study [by Gruzdyn et al. (139)] and to further investigate the inhibitory impact of TME-derived IL-10 on CAR-T therapy, Batchu and colleagues conducted a study and reported that blockade of IL-10 in the tumor milieu remarkably reverses its pro-tumor effects and enables mesothelin-redirected CAR-Ts to more efficiently exert their tumoricidal effects (140). Briefly, these researchers developed mesothelin-redirected CAR-Ts and prepared a conditioned medium from two-day cultivation of SKOV3 cells without serum in which IL-10 was present or depleted via antibodies (referred to as IL-10-proficient or IL-10-deficient medium, respectively) (140). Cultivation of mesothelin-redirected CAR-Ts in the IL-10-proficient media resulted in significantly suppressed secretion of granzyme B and INF-γ, as their secretion levels were not completely returned to the co-cultivation levels even in the presence of IL-10-deficient media (140). Moreover, a sharp decline in the cytolytic reactions of mesothelin-redirected CAR-Ts was reported (to 19% at the E:T ratio of 10:1 and 32% at the E:T ratio of 20:1) in the presence of IL-10-proficient media (140).

Simultaneous expression of CAR-T-targeted TAAs by healthy tissues results in off-tumor toxicities against unintended tissues, to overcome which, some researchers have suggested the development of mRNA-based CAR-Ts that transiently express CARs redirected against TAAs of interest (60, 141). According to a study by Hung and colleagues, the researchers generated an automated and efficient platform for the large-scale development (~ 2 × 10^10^) of mRNA-based human mesothelin-redirected CAR-Ts that enables the generation of vast populations of CAR-Ts from a single round of leukapheresis to be used for multiple infusions into treatment subjects (141). According to *in vitro* killing assays, the developed CAR-Ts mediated strong tumoricidal effects against the murine ovarian cancer cell line Defb29 engineered to express human mesothelin, as it was reported that CAR expression rate grew low within a week following *in vitro* cultivation with a possible correlation with CAR-T expansion (141). Moreover, the investigators established human mesothelin-positive ovarian tumor mouse models (using 3 × 10^5^ human mesothelin-positive Defb29 cells), and reported that a single round intraperitoneal administration of mesothelin-redirected CAR-Ts resulted in tumor outgrowth suppression and prolonged survival of the animal models in a CAR-T dose-dependent fashion (141). The researchers also reported that repeated weekly intraperitoneal injection of an optimal mesothelin-redirected CAR-T dose resulted in better tumor regression and more protracted survival rates in the mouse models (141). Of note, no serious CAR-T-mediated off-tumor toxicities were reported by the investigators (141). Such findings highlight the potential applicability of mRNA-based mesothelin-redirected CAR-Ts for clinical evaluations in individuals with mesothelin-positive ovarian tumors, as well as other relatable malignancies. According to another study, Haas and colleagues reported the findings of a Phase I clinical investigation that evaluated the safety and efficacy of mesothelin-redirected CAR-Ts in fifteen individuals with ovarian cancer, pancreatic adenocarcinoma, and pleural mesothelioma (five patients in each oncological indication group; who were also refractory to chemotherapy) (142). Briefly, lentivirally transduced CAR-Ts were generated by incorporating a mesothelin-specific scFv (derived from the murine mAb, SS1) into the construct of a 2^nd^-generation CAR (based on 4-1BB and CD3ζ as the co-stimulatory domain and activation domain, respectively), and then the patients underwent a single round of CAR-T administration (with 1-3 × 10^7^ or 1-3 × 10^8^ engineered effector cells/m^2^) with cyclophosphamide-induced lymphodepletion (1.5 g/m^2^) or without lymphodepletion (142). Conclusively, it was reported that the CAR-Ts were well-tolerated, as only one case of toxicity was reported (grade 4) in a non-lymphodepleted patient in the low-dose CAR-T group (142). Eleven out of fifteen (~ 73%) patients achieved stable disease, as CAR-T persistence was reported to be transient with their expansion peak between day 6 to 14 (of note, prior lymphodepletion improved effector cell expansion, but had no positive effects on their persistence after 28 days) (142). According to the blood examination results of fourteen patients, neutralizing antibodies against the CAR constructs of the CAR-Ts were found in eight patients (~ 57%), which necessitates the application of fully human or humanized antigen recognition domains (scFvs or VHHs) in the design of CAR constructs for clinical applications (60, 80, 142, 143).

According to another study, Zhang and colleagues focused on targeting two distinct epitopes of mesothelin (its membrane-distal region and membrane proximal region; hereinafter referred to as meso-I and meso-III, respectively) by developing two different mesothelin-redirected CAR-T products (144). For *in vitro* analysis of the antitumor reactivities of the developed CAR-T products, meso-I- or meso-III-redirected CAR-Ts were co-cultivated with the human gastric carcinoma cell line HGC-27 or human ovarian cancer cell line SKOV3, and it was demonstrated that meso-III-redirected CAR-Ts exhibited more pronounced tumoricidal effects against the target cells, as they also secreted higher levels of IL-2, INF-γ, and TNF-α, and demonstrated a higher expression rate of CD107α in comparison with those of meso-I-redirected CAR-Ts (144). Furthermore, Zhang et al. investigated the antitumor efficacy of the developed CAR-Ts in more realistic tumor models of the HGC-27 and SKOV3 cell lines by developing 3D tumor spheroids and reported that meso-III-redirected CAR-Ts cytolyzed higher percentages of the target cells over a period of 24 hours (144). In mouse models of HGC-27-based gastric cancer, meso-III-redirected CAR-Ts, which were administered intravenously ten days following tumor establishment, enforced more effective tumoricidal effects which resulted in more pronounced tumor volume shrinkage in the animal models, as compared to those of meso-I-redirected CAR-Ts (144). Moreover, in mouse models of SKOV3-based ovarian cancer, meso-III-redirected CAR-Ts were administered intravenously seven days or fourteen days following tumor establishment, and it was demonstrated that the CAR-Ts were able to mediate similar survival rates (over the course of 40 days) and similar tumor volume shrinkage in the treatment subjects (144). Based on these findings, it can be concluded that meticulous selection of the target antigen epitope could be a factor of paramount importance in the context of CAR-T therapy development, which could potentially lead to better therapeutic effects.

According to a 2021 report, Liu and colleagues conducted an investigation to assess the inhibitory effects of PD1-PD-L1 interaction on the tumoricidal effects of mesothelin-redirected CAR-Ts, using the SKOV3 and HCT116 (of colorectal cancer origin) cell lines and a shRNA-based silencing strategy for PD-1 silencing (145). According to the results of the *in vitro* experiments, PD-1-silenced mesothelin-redirected CAR-Ts exhibited stronger tumoricidal effects against the SKOV3 and HCT116 cell lines upon co-cultivation, alongside secreting higher levels of INF-γ, in comparison with those of wild-type mesothelin-redirected CAR-Ts (145). Such findings highlight the importance of PD1-PD-L1 axis disruption in the context of solid tumor CAR-T therapy which warrants meticulous in-depth preclinical and clinical evaluations (145). Moreover, other gene silencing techniques (such as CRISPR-Cas or TALEN) alongside other immunoinhibitory genes can also be taken into consideration for future assessments (24, 102, 145). Another investigation further investigated how the immunosuppressive elements of ovarian tumors impede the functionality of CAR-Ts and demonstrated that the co-stimulatory domains of CAR constructs could play important roles in this matter (146). Briefly, Schoutrop and colleagues developed two different mesothelin-redirected CAR-T products, one of which harbored the CD28 co-stimulatory domain whereas the other had the 4-1BB co-stimulatory domain, and evaluated the *in vivo* functionality of these CAR-Ts in SKOV3-based xenograft mouse models of ovarian cancer (146). Briefly, adoptive transfer of CD28-based CAR-Ts resulted in significant protracted survival of the animal models whereas 4-1BB-based CAR-T treatment mediated persistent remission in several treatment subjects (146). Moreover, examination of tumor-infiltrating CAR-Ts demonstrated that both CAR-T products exhibited elevated levels of immunoinhibitory receptor (LAG3 and PD-1) expression which coincided with the expression of relative ligands (HLA-DR and PD-L1) by mesothelin-positive ovarian tumors (146). However, it was demonstrated that tumor-infiltrating CD28-based CAR-Ts exhibited more pronounced characteristics of exhaustion in comparison with 4-1BB-based CAR-Ts (146). Such findings substantiated the proposition that immunoinhibitory axes impair the persistence of CAR-Ts in the TME of ovarian cancer (146).

Most patients with solid tumors, such as ovarian cancer, often suffer from tumor recurrence which is somehow a result of antigen heterogeneity within the TME (43, 147). In the context of mesothelin-redirected CAR-T therapy for ovarian cancer, a recent study by Liang and colleagues attempted to address this issue through the development of tandem CAR-Ts that target FOLR1 (by the means of the *MOv19* scFv) and mesothelin (by the means of the *P4* scFv) while engineered to secrete IL-12 for supportive immunomodulatory effects (147). Briefly, these researchers put together a panel of twenty co-expressed genes with elevated expression levels using the Gene Expression Omnibus, and then selected FOLR1 and mesothelin as two co-expressed potential target antigens, as only a small percentage (~ 10%) of the sample were deficient in their co-expression (147). FOLR1-redirected CAR-Ts, mesothelin-redirected CAR-Ts, and tandem CAR-Ts were co-cultivated with the SNU119 and SKOV3 ovarian cancer cell lines, and it was demonstrated that tandem CAR-Ts mediated higher percentages of target cell lysis in comparison with those of the single antigen-redirected CAR-Ts or control T cells (147). Moreover, significantly higher levels of IL-2, IL-12, INF-γ, and TNF-α were secreted by tandem CAR-Ts upon their co-cultivation with the SNU119 cells, in comparison with those of other CAR-Ts or T cell groups (147). These researchers further developed ovarian cancer xenograft models based on B-NDG mice and SNU119 cells and treated the animal models with intravenous administration of the three different CAR-T products or control T cells (1 × 10^7^ cells) ten days following tumor transplantation (147). Tandem CAR-T significantly outperformed other treatment groups in terms of mediating prolonged survival and eliminating large established ovarian tumor lesions; however, tandem CAR-Ts exhibited a comparable persistence rate and infiltration capacity only to FOLR1-redirected CAR-Ts, which was higher than those of the other CAR-T/T cell groups (147). Ultimately, Liang and colleagues concluded that FOLR1/mesothelin tandem CAR-Ts engineered to secrete IL-12 could minimize the risks of disease recurrence through the elimination of antigen escape variants while maximizing targeted antitumor responses (147). However, broad clinical investigation must elucidate the safety and tolerability of such CAR-T platforms, since the selection of two co-expressed target antigens absent from healthy tissues could be a rather challenging task (102).

According to a 2021 report by Liu and colleagues, it was reported that disruption of adenosine 2a receptor (A2aR) culminates in improved tumoricidal efficacy of mesothelin-redirected CAR-Ts in preclinical conditions (148). Accumulating evidence demonstrates that tumor cell-derived adenosine, within the TME, binds its cognate receptor, A2aR, on the surface of T cells leading to their impaired tumoricidal reactivity through triggering downstream signaling cascades (148). To evaluate the effects of A2aR disruption on the tumoricidal efficacy of mesothelin-redirected CAR-Ts, Liu et al. used a specific shRNA for A2aR disruption (148). Briefly, it was demonstrated that A2aR-disrupted mesothelin-redirected CAR-Ts outperformed conventional mesothelin-redirected CAR-Ts in terms of mediating cytolytic reactions and secreting proinflammatory cytokines upon their co-cultivation with the SKOV3 and HCT116 cell lines (148). Moreover, adoptive transfer of both mesothelin-redirected CAR-T products into SKOV3-based xenograft mouse models resulted in remarkable tumor regression, as compared with the control T cell group, with the A2aR-disrupted CAR-Ts mediating more pronounced tumoricidal reactions (148). Conclusively, these researchers asserted that shRNA-based gene disruption might hold therapeutic promise for augmenting the antitumor efficacy of CAR-Ts within harsh TME conditions (148). The therapeutic benefit and clinical feasibility of this strategy need to be meticulously assessed in clinical investigations with ovarian cancer patients.

Aside from the encouraging results of 2^nd^-generation mesothelin-redirected CAR-Ts, their 3^rd^-generation counterparts could also be therapeutically valuable in the context of ovarian cancer. According to a 2021 report, Zhang and colleagues incorporated a mesothelin-specific scFv into a 3^rd^-generation CAR construct based on the CD28/4-1BB co-stimulatory domains and the CD3ζ activation domain only to develop mesothelin-redirected CAR-Ts (149). Upon co-cultivation of these CAR-Ts with the SKOV3 and OVCAR3 ovarian cancer cell lines, the effector cells mediated mesothelin-dependent tumoricidal effects against the target cells (149). Moreover, the CAR-Ts secreted significantly elevated levels of INF-γ and TNF-α upon co-cultivation with the SKOV3, OVCAR3, and HCT116 cell lines (149). To further evaluate the antitumor efficacy of these mesothelin-redirected CAR-Ts, Zhang et al. established mouse models of ovarian cancer, breast cancer, and colorectal cancer (by subcutaneous injection of SKOV3, MCF7, and HCT116 cells into the animal models, respectively), and reported that intravenous administration of the mesothelin-redirected CAR-Ts (2.5 × 10^5^ effector cells) resulted in significant shrinkage of the established tumor lesions (149). Moreover, the adoptive transfer of the mesothelin-redirected CAR-Ts into mesothelin-positive patient-derived xenograft mouse models of colorectal cancer or gastric cancer resulted in the eradication of the tumor bulks and prolonged survival of the animal models (149). Such findings highlighted the fact that 3^rd^-generation mesothelin-redirected CAR-Ts could also be choices of therapeutic value against ovarian cancer in preclinical settings; however, clinical studies must be conducted to directly assess the safety profile and tumoricidal efficacy of 2^nd^-generation and 3^rd^-generation mesothelin-redirected CAR-Ts in patients with mesothelin-positive ovarian cancer (149). In 2022, Li and colleagues incorporated the humanized version of a mesothelin-specific single-domain antibody (known as *F3M*) into a 2^nd^-generation CAR construct based on the 4-1BB and CD3ζ co-stimulatory domain and activation domain, respectively, and developed mesothelin-redirected CAR-Ts that exhibited effective tumoricidal efficacy in preclinical settings (150). As an attempt to counteract the immunosuppressive effects of TME-derived TGF-β, Li and colleagues engineered T cells to co-express the mentioned CAR alongside a dominant-negative TGF-β receptor type II (150). Li et al. demonstrated that the developed CAR-Ts exhibited ameliorated efficacy in the presence of TGF-β and in preclinical mouse models, in a way that these CAR-Ts were capable of resisting TGF-β immunosuppressive effects (150). In October 2020, a Phase I clinical investigation was initiated to investigate the safety and tumoricidal efficacy of these CAR-Ts in fifteen patients with ovarian cancer; however, in 2022, the collaborators and sponsors were considering bringing closure to the investigation.

To address one of the most important obstacles of solid tumor CAR-T therapy, which is poor CAR-T trafficking into the tumor sites, Pang and colleagues developed glypican 3- or mesothelin-redirected CAR-Ts engineered to express and secrete IL-7 and CCL19 and demonstrated that these CAR-Ts exhibited enhanced migration capacity and expansion rate *in vitro* (151). Previous studies have demonstrated that IL-7 and CCL19 expression correlates with an enhanced infiltration rate of T cells in preclinical experiments (42, 151, 152). Moreover, these researchers reported that adoptive transfer of their IL-7/CCL19-engineered CAR-Ts mediated strong tumor regression in xenograft models of liver cancer and pancreatic cancer (151). Relying on these findings, Pang et al. initiated a Phase I clinical investigation to evaluate the safety and applicability of these CAR-Ts in individuals with ovarian cancer (proficient in the expression of glypican 3 or mesothelin), as well as other advanced solid tumors (NCT03198546) (151). The results of this trial relating to the treatment of ovarian cancer patients are yet to be obtained; however, these researchers reported that intratumoral administration of the IL-7/CCL19-engineered glypican 3-redirected CAR-Ts led to complete tumor eradication a month following the treatment in a patient with hepatocellular carcinoma (151). In reference to IL-7/CCL19-engineered mesothelin-redirected CAR-Ts, it was reported that the adoptive transfer of these CAR-Ts to a pancreatic cancer patient led to the complete elimination of tumors almost 8 months following CAR-T treatment (151). Ultimately, it can be concluded that engineering CAR-Ts for the secretion of IL-7 and CCL19 could have a therapeutic advantage against solid tumors by increasing the tumor-homing capacity of CAR-Ts; however, in the context of ovarian cancer, clinical investigations with large patient populations must be conducted for more elucidation (151). In 2022, Chen and colleagues isolated a mesothelin-specific scFv without any cross-reactivity using the phage-display technology and then developed 2^nd^-generation CAR-Ts (153). Following successful *in vitro* and *in vivo* antitumor functionality of these mesothelin-redirected CAR-Ts against ovarian cancer cell lines and xenograft mouse models, respectively, Chen and colleagues evaluated the safety profile and therapeutic efficacy of autologous mesothelin-redirected CAR-Ts in three individuals with ovarian carcinoma (153). No CRS nor neurotoxicity (greater than grade 2) was reported in any of the three patients, as two of them experienced stable disease with a PFS of 4.6 and 5.8 months (153).

In 2022, Tanyi and colleagues reported the results of a Phase I clinical investigation in which fourteen patients with mesothelin-positive solid tumors, inclusive of ovarian cancer, underwent treatment with mesothelin-redirected CAR-Ts harboring a humanized scFv as the targeting domain incorporated into a 4-1BB/CD3ζ-based CAR construct (154). The patients were put into four different cohorts (1 to 4); three patients were treated with a single intravenous administration of 3 × 10^7^ CAR-Ts/m^2^, three underwent pre-treatment lymphodepletion and a single intravenous administration of 3 × 10^7^ CAR-Ts/m^2^, two underwent a single intravenous administration of 3 × 10^8^ CAR-Ts/m^2^, and six underwent lymphodepletion and an initial intravenous administration of 3 × 10^7^ CAR-Ts/m^2^ scheduled to be followed by a maximum of two other intravenous infusions (cohort 1, 2, 3, and 4, respectively) (154). Briefly, the treatment was reported to be well-tolerated, as CAR-T expansion directly correlated with lymphodepletion and higher infusion doses (154). Moreover, the administered CAR-Ts exhibited tumor infiltration in nine out of fourteen patients (~ 64%) (154). In accordance with the RECIST criteria, eight patients experienced stable disease (~ 57%); which lasted for three and nine months in two of the patients (154). In reference to the adverse events and toxicities, CRS (grades 3 and 4) was documented in four patients, as multiple adverse events were also observed (which included hypotension, fatigue, and hypoxia) (154). Serious pulmonary-related toxicities were only documented in the patients of cohort 3; one of whom experienced failure of the respiratory system, which was related to the high dosage of the administered CAR-Ts (154). The instigators declared 3 × 10^7^ CAR-Ts/m^2^ as the maximum tolerated dose based on the findings of this trial (154). Ultimately, Tanyi and colleagues asserted that these findings could pave the way for future clinical trials assessing the safety and therapeutic applicability of CAR-Ts in mesothelin-positive tumors, including ovarian cancer, and that meticulous strategies could be taken into consideration for increasing the safety profile and tumor-homing capacity of such CAR-Ts (such as localized CAR-Ts administration) (154). Studies such as those discussed in this section highlight the importance of mesothelin as a potential target antigen in ovarian cancer CAR-T therapy, and the fact that researchers have put a tremendous deal of effort into assessing CAR-T therapy in the treatment of mesothelin-positive ovarian cancer; however, the findings of future clinical trials with larger patient populations could shed more light on the downsides and upsides of ovarian cancer CAR-T therapy, and propose applicable strategies for overcoming the limitations.

## 5 Conclusion

Reaching the destination is worth having a long and twisted way to go; this might be the case with CAR-T therapy in the treatment of ovarian cancer. Researchers must keep in mind that numerous dots must be connected to successfully fight this advanced indication with CAR-Ts. Over the past years, tremendous effort has been put into assessing the safety and therapeutic potential of CAR-T therapy redirected against various ovarian cancer-associated target antigens both in preclinical and clinical investigations. To fully validate the suitability of each of these target antigens in the context of ovarian cancer CAR-based therapies, broad and in-depth clinical findings are required. However, other platforms of cancer immunotherapy have been developed against some of these antigens, as well as other target antigens whose targeted therapy might hold therapeutic promise. For instance, ImmunoGen developed a FRα-specific ADC named mirvetuximab soravtansine (under the trade name Elahere^®^) which was approved by the US FDA in November 2022 for the treatment of patients with epithelial ovarian cancer resistant to platinum or peritoneal cancer with three or fewer lines of prior therapies (155). The approval of this therapeutic was based on the ultimate findings of a clinical trial (NCT04296890) in which 106 patients with the mentioned indications were required to undergo bevacizumab. The objective response rate (ORR) and duration of response (DOR) were reported to be ~ 32% and 6.9 months, respectively. Moreover, this therapeutic entered the market with a safety label for ocular toxicity; meaning that despite encouraging clinical outcomes, there are still risks of off-tumor toxicities. The same assumption could be attributed to all of the antigens discussed throughout this review, as CAR-T therapies and mAbs exert their antitumor function through distinct mechanisms. In brief, mAbs can play an antagonistic role by blocking ligands from binding their cognate receptors, induce signaling in a cell upon antigen engagement, or trigger target cell lysis through complement-dependent cytotoxicity (CDC) or antibody-dependent cellular cytotoxicity (ADCC). In contrast, while CAR-Ts engage with their target cells upon antigen encounter, they mediate cytolytic reactions against that cell, which might result in serious irreversible organ damage in the cases of off-tumor effects. Therefore, it is reasonable to assume that CAR-Ts have different toxicity profiles in comparison with those of mAbs, or any other targeted treatment modality, and antigens considered suitable for mAb therapies might not necessarily be suitable for CAR-T therapy as well. Another justification might be based on the lack of qualified targeting domains for the construction of potent CARs against such targets (which might arise from proprietary rights). Ultimately, since CAR-T therapy of ovarian is still a progressing field, it is believed that the suitability of these antigens for CAR-T therapy of ovarian cancer will be a subject of investigation in the upcoming years (as some of these antigens have already been assessed in the case of other solid tumors). Out of the target antigens discussed, three mAbs against PD-L1 have been approved by the US FDA which include durvalumab (IMFINZI^®^; approved for the treatment of bladder cancer in 2017), avelumab (Bavencio^®^; approved for the treatment of Merkel cell carcinoma in 2017), atezolizumab (Tecentriq^®^; approved for the treatment of bladder cancer in 2016) (156–158). These approvals might somehow corroborate the suitability of PD-L1 as a target antigen of cancer immunotherapy; however, each immunotherapy platform warrants further in-depth assessments of its own.

Aside from selecting the ideal target antigen, successful CAR-T therapy in solid tumors including ovarian cancer depends on various factors that are critical for tackling the roadblocks of this type of treatment. The future of this type of therapy relies on the engineering of next-generation CAR-Ts and their successful employment. For instance, since most of the targeted antigens in the CAR-T therapy of ovarian cancer are TAAs, researchers need to develop strategies to overcome the issue of on-target off-tumor toxicities. One of the most applicable strategies in this matter is the use of CAR targeting domains with a moderate affinity, rather than high-affinity ones (159). Experimental findings have demonstrated that this strategy is feasible in minimizing the off-tumor effects of CAR-Ts on healthy tissues (159). Other strategies could be based on the transient expression of CAR molecules in CAR-Ts developed using mRNAs or the use of suicide switches for the elimination of the infused T cells (160, 161). In the context of ovarian cancer CAR-T therapy, various researchers reported the presence of neutralizing antibodies against the targeting domain of CARs derived from animal-based targeting moieties. Such antigen-recognition domains must be replaced with humanized or fully human targeting domains to overcome the issue of anaphylaxis (60, 80). As discussed, the design of the CAR molecules (the spacer fragment, the targeting domain, and the signaling domains) could have substantial effects on the phenotype and antitumor efficacy of the developed CAR-Ts; therefore, precise designing alongside meticulous selection of the CAR components should be taken into consideration before the development of a CAR-T product. Researchers also used an intelligent strategy to overcome the issue of low antigen density by ovarian cancer cells. As detailed throughout the text, various therapeutics (such as chemotherapeutics) were used to positively influence the expression of a certain antigen by tumor cells; thereby increasing their susceptibility to CAR-T-mediated cytolytic reactions. To overcome the immunosuppressive nature of the TME of ovarian cancer, CAR-Ts could be genetically engineered to be immunosuppression-resistant (using CRISPR-Cas, TALEN, shRNAs, etc.) or secrete anti-immunoinhibitory molecules (such as anti-PD-1 scFvs or VHHs) (26). To overcome the limitation of poor CAR-T infiltration in the context of ovarian cancer, localized administration of CAR-Ts could be a potent solution instead of systemic administration. Moreover, CAR-Ts could also be genetically modified for the expression of chemokine receptors that correspond to those secreted by tumor cells for amplifying the presence of effector immune cells in tumor sites. For instance, it has been demonstrated that CAR-Ts can be genetically engineered for the expression of IL-8 receptors (CXCR1 or CXCR2) (27, 162). Such CAR-Ts can respond to IL-8 secreted by tumor cells in tumor islets which results in boosted CAR-T infiltration rate and improved antitumor efficacy (27, 162). To solve the issue of tumor escape variants or tumor heterogeneity, the modification of CAR-Ts for the expression of molecules that engage the endogenous elements of the immune system (such as IL-12) or the co-expression of distinct CAR molecules that target two (or more) sets of target antigens associated with ovarian cancer could be taken into consideration. Aside from all of the discussed engineering novelties, CAR-Ts can be designed to trigger bystander antitumor activity by other components of the immune system. For instance, CAR-Ts can be engineered to secret TRBAs which can help them circumvent antigen escape and induce paracrine immune responses by bystander T cells against the tumor cells (163). Similarly, CAR-T can also be engineered to express CD40 ligand (CD40L) on their surface which can act through two mechanisms. In detail, CD40L-expressing CAR-Ts can interact with CD40-expressing tumor cells which results in a direct antitumor effect. Also, CD40L-expressing CAR-Ts have the ability to license antigen-presenting cells (APCs) that can mediate the recruitment and cytokine production of other endogenous immune effector cells (164, 165). Moreover, allogeneic CAR-Ts have been used in clinical settings owing to their various advantages (166). However, the limitation of alloreactivity of these products should be addressed to avoid compromised antitumor responses in patients. To address this, researchers have used genetic manipulation methods to knock out the TCR α or β chain which renders the produced T-cell product devoid of TCR surface presentation resulting in a significantly lower rate of alloreactivity (167–171). Moreover, similar methods have also been applied for the knockout of human leukocyte antigens (HLA) expression in T-cell-based products to minimize their alloreactivity and expand their safer clinical application (172, 173).

In the long run, it can cautiously be concluded that considerable preclinical and clinical efforts have been made in the field of ovarian cancer CAR-T therapy. However, how well-tolerated CAR-T targeting of each of these antigens will be in patients with ovarian cancer is the subject of future investigations, as limitations currently known or unknowns will have to be overcome. The future success in this field is highly dependent on the specificity and safety of the target antigens as well as the counterstrategies for tackling the roadblocks of this type of solid tumor. In a similar fashion to CAR-T therapy in most types of solid tumors, the application of this type of living drug immunotherapy in ovarian cancer requires far-reaching detailed investigations, especially in clinical settings with larger patient populations.

## Author contributions

FN: Investigation, Validation, Writing – original draft. KF: Validation, Writing – original draft. PouSK: Conceptualization, Investigation, Supervision, Validation, Visualization, Writing – original draft, Writing – review and editing. MMK: Writing – original draft. SDS: Investigation, Writing – original draft. PooSK: Conceptualization, Investigation, Supervision, Validation, Visualization, Writing – original draft, Writing – review and editing.
